# Role of Technology Flexibility and Grid Coupling on Hydrogen Deployment in Net-Zero Energy Systems

**DOI:** 10.1021/acs.est.4c12166

**Published:** 2025-03-04

**Authors:** Jun Wen Law, Bryan K. Mignone, Dharik S. Mallapragada

**Affiliations:** † MIT Energy Initiative, Massachusetts Institute of Technology, Cambridge, Massachusetts 02139, United States; ‡ ExxonMobil Technology and Engineering Company, Annandale, New Jersey 08801, United States; § Chemical and Biomolecular Engineering Department, Tandon School of Engineering, New York University, Brooklyn, New York 11201, United States

**Keywords:** energy system decarbonization, macro energy system modeling, hydrogen supply chain, electrolysis, blue hydrogen, CO_2_ removal

## Abstract

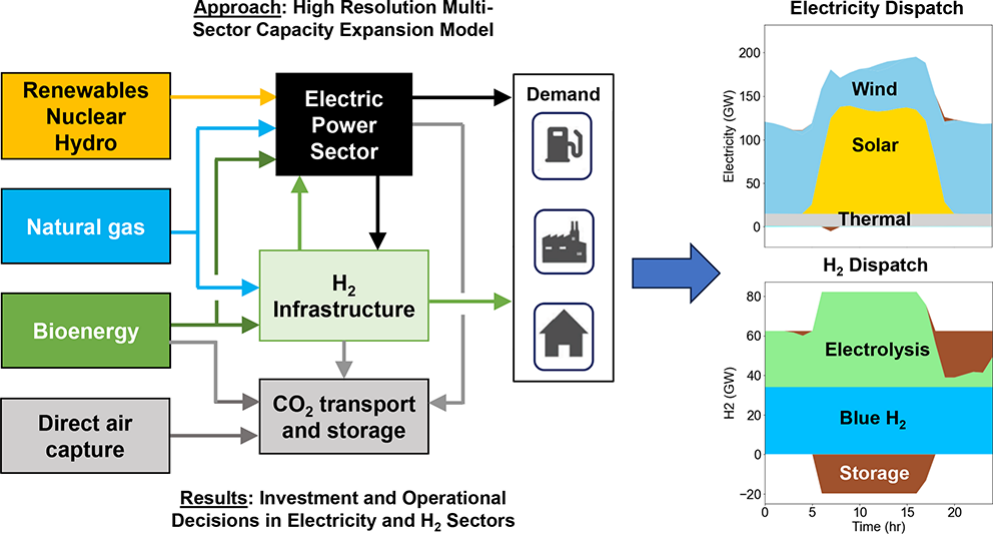

Low-carbon hydrogen is anticipated to be a key element of economy-wide decarbonization pathways. Here we employ a multisector energy system model of the contiguous United States to study competition among low-carbon hydrogen production options and the interplay between the electricity and hydrogen sectors in a net-zero energy system. When hydrogen storage is available without constraints and electrolyzers are grid-connected, they account for most hydrogen production, while providing demand-side flexibility to the electricity system. This decreases battery storage deployment to achieve similar shares of variable renewable energy (VRE) in the power system. When electrolyzers are not grid-connected but rely on islanded VRE power to produce “green” H_2_, we find that power system flexibility and the share of electrolytic hydrogen are reduced, all else equal. Without hydrogen storage, natural gas-based hydrogen (i.e., “blue” H_2_) accounts for most hydrogen production, although increasing flexibility of blue H_2_ can enable some electrolytic H_2_ production. Finally, we find that hydrogen deployment does not substantially drive energy transmission expansion, although there is a modest increase in CO_2_ transmission when blue H_2_ is deployed in regions with limited CO_2_ storage.

## Abstract 2

Using a multisector energy system model, this study examines the drivers of electrolytic and blue hydrogen deployment in net-zero emissions systems.

## 1 Introduction

As the emphasis of regional and national decarbonization moves beyond the power sector, there is growing interest in hydrogen (H_2_) as a decarbonization solution for end uses in which direct electrification may be difficult or costly, such as in some parts of the industrial and transport sectors. Part of the appeal of hydrogen is its versatility, as it can be produced from different feedstocks and production routes and potentially consumed in several different end uses. However, the number of potential pathways adds analytical complexity, which is further compounded by the cross-sectoral interactions implied by each pathway. For example, electrolytic H_2_ production, often referred to as “green H_2_” when electricity is sourced from variable renewable energy (VRE), directly impacts the electric power sector by increasing aggregate electricity demand and changing when that demand occurs. Further, as highlighted by many recent studies,^1−3^ the emissions associated with grid-connected electrolytic H_2_ are not straightforward to quantify due to their spatial and temporal variability.

Cross-sectoral interactions are not restricted to those related to electrolytic H_2_, but extend to other low-carbon hydrogen production pathways including natural gas-based hydrogen production with carbon capture and sequestration (CCS), often called “blue H_2_,” and biomass-based production with CCS (BECCS H_2_). For both production routes, the captured CO_2_ requires additional investment in CO_2_ pipelines to connect production with CO_2_ sequestration sites, and these costs could be shared with or affected by CCS deployment in other sectors. In addition, for natural gas routes, any residual emissions would need to be offset by deployment of negative emissions technologies such as BECCS or direct air capture (DAC) with CO_2_ storage in a net-zero CO_2_ emissions system. Deployment of BECCS or DAC, in turn, could further affect the electricity or hydrogen production sectors.

Several prior studies have undertaken techno-economic analysis (TEA) and life cycle analysis (LCA) of electrolytic and blue H_2_ with a focus on quantifying the levelized cost of hydrogen (LCOH) based on assumed electricity prices and CO_2_ costs after accounting for the life cycle carbon intensity of electricity supply.^4−8^ While such studies examine technologies at a relatively granular process level to estimate the cost of deploying electrolytic or blue H_2_, this type of static approach does not consider drivers related to system integration. For example, TEA studies of grid-powered electrolytic H_2_ commonly assume a capacity factor for electrolyzers without explicitly representing the temporal variability of electricity supply or the operational flexibility of electrolyzers, which could alter the economics and emissions of electrolysis as well as electricity production.

To partially address this shortcoming, some studies^9−11^ have optimized electrolyzer operation subject to an exogenous time profile of electricity supply or prices. However, assuming electrolyzers are price-takers does not consider how their deployment may affect the operation of the electricity system and thus how co-optimization of the two sectors may affect technology choice. This lack of explicit sectoral coupling is also a limitation of TEA of natural gas-based routes, since the additional abatement cost of residual emissions is not linked to deployment of negative emissions technologies (NETs) within the energy system. As such, this approach omits any potential impacts of deploying NETs in the electricity and hydrogen production sectors that could be relevant to technology selection in these sectors.

An alternative approach uses multisector capacity expansion models (CEMs) to evaluate the cost-optimal composition and operation of an energy system subject to several technological and system-level constraints. Such energy system models are increasingly used for deep decarbonization analysis of various regions and can shed light on competition between technologies under a range of technology, market, and policy assumptions.^12−14^ However, such approaches are often unable to include as much technological granularity as TEA and LCA due to the need to manage the computational burden that arises from a considerably wider sectoral and technological scope combined with high temporal resolution.

Prior studies using multisector CEMs to examine hydrogen have tended to focus on coupling electrolytic H_2_ with the power sector and on the implications for electrolytic H_2_ production and hydrogen-based power generation.^15−22^ In many instances the competition between electrolytic and other low-carbon hydrogen technologies and the potential roles for other complementary technologies such as NETs was not considered.^20,23^ Other multisector CEM studies have focused on identifying the role of hydrogen in regional decarbonization pathways but paid less attention to the competition between electrolytic and blue H_2_ or the factors affecting this competition.^12,14^ Moreover, many of the studies did not explicitly examine the sensitivity to one or more of the following assumptions: availability of NETs, availability of H_2_ storage and pipelines, blue H_2_ operational flexibility, or whether electrolytic H_2_ production relies on the electricity grid or on a self-contained (“islanded”) renewable electricity system.^2,15,24−27^ Islanded systems are increasingly being considered to avoid delays or challenges in connecting electrolyzers to the grid and to simplify carbon emissions accounting.^28^

This study addresses key gaps in prior work by examining technology choice in the hydrogen production sector using a coupled electricity-hydrogen system model that includes a representation of NETs, operational flexibility of hydrogen production technologies, and hydrogen storage and pipeline infrastructure (which we refer to as “infrastructure” for brevity). We focus on how various forms of technological flexibility affect potential outcomes for the contiguous United States in 2050 under a net-zero emissions constraint. We find that flexibility provided by hydrogen infrastructure (primarily hydrogen storage) strongly affects the choice between electrolytic and blue H_2_, with assumptions about the availability of NETs and blue H_2_ operational flexibility also affecting some outcomes. In addition, we find that electrolyzers can provide flexibility to the grid, which may affect electricity system technology choices and, in turn, the competitiveness of grid-connected electrolysis relative to islanded electrolytic production.

The remainder of this paper is organized as follows. Section 2 describes the modeling approach, assumptions, and scenarios. Section 3 describes the key findings from the scenarios evaluated. Section 4 concludes by discussing the implications of these results and potential areas for future work.

## 2 Materials and Methods

### 2.1 MACRO Model

**Figure 1:**
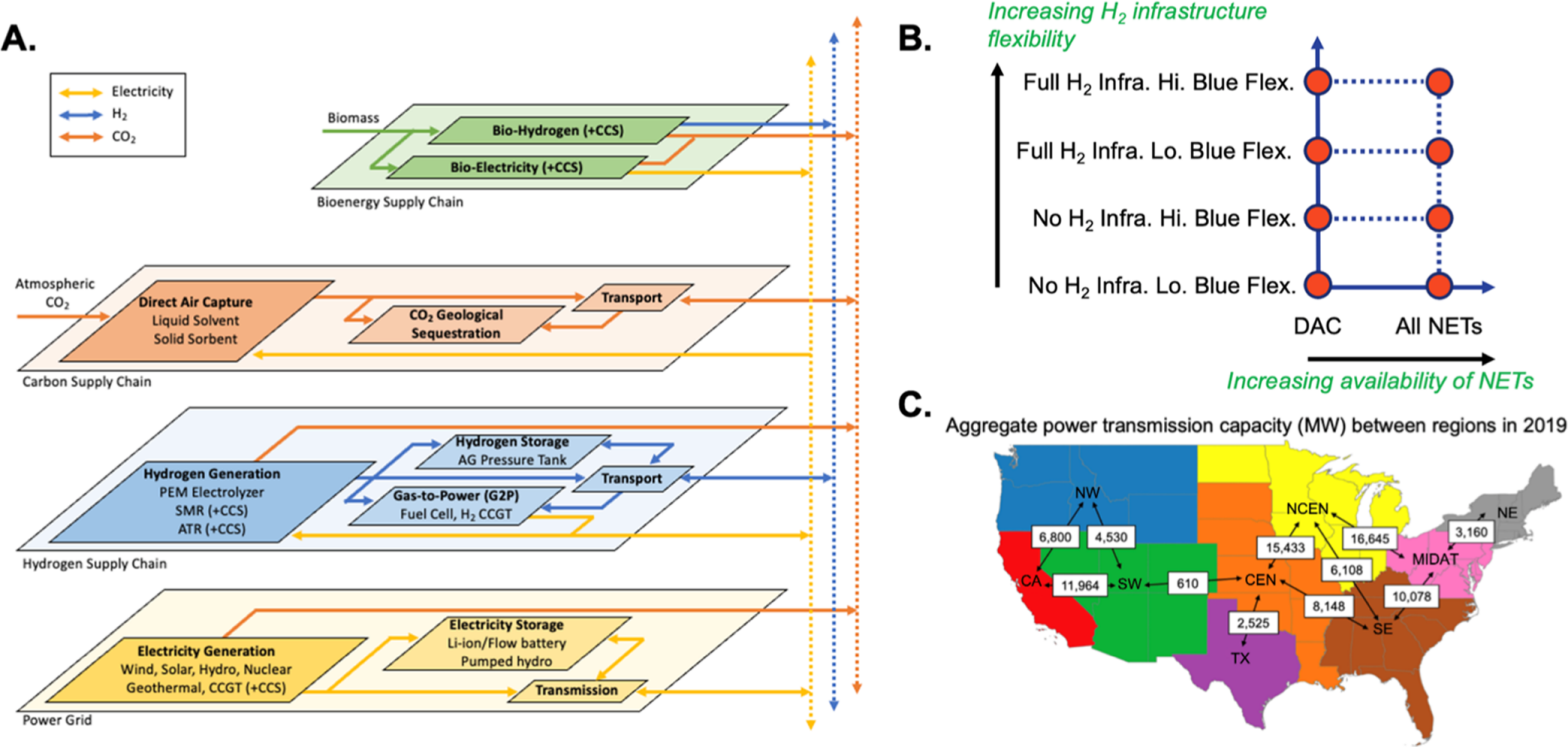
(A) Architecture of the coupled MACRO energy system. (B) Scenario matrix showing eight core scenarios evaluated in this study developed by varying assumptions about H_2_ infrastructure flexibility and NETs availability. “Full H_2_ Infra.” = Availability of H_2_ storage and H_2_ pipelines, “No H_2_ Infra.” = No H_2_ storage and H_2_ pipelines, “Hi. Blue Flex.” = Blue H_2_ plants operate between 50 and 90% of nameplate capacity; “Lo. Blue Flex.” = Blue H_2_ plants operate between 85% and 90% of nameplate capacity, “DAC” = Only direct air capture (DAC) can provide negative emissions, “All NETs” = DAC and bioenergy with CO_2_ capture and sequestration (BECCS) in electricity and hydrogen production can provide negative emissions. (C) Nine representative regions of the contiguous U.S. based on the Open Energy Outlook 2022,^14^ with aggregated initial power transmission capacity between regions obtained from the U.S. Environmental Protection Agency (EPA) version of the Integrated Planning Model (IPM).^30^ California (“CA”), Northwest (“NW”), Southwest (“SW”), Texas (“TX”), North Central (“NCEN”), Central (“CEN”), Southeast (“SE”), Mid-Atlantic (“MIDAT”), Northeast (“NE”). Blue H_2_ plants include steam methane reforming and autothermal reforming with carbon capture and storage (CCS) as shown in Table S13.

The open-source MACRO model is formulated as a multisector capacity expansion model that evaluates least-cost investment and operation decisions across the supply chains of electricity, hydrogen, captured CO_2_, and bioenergy for a future year.^29^Figure 1A shows the architecture of MACRO, which allows for representation of a wide range of technology options across sectors, including production, storage, and transmission technologies, as well as NETs.

In MACRO, electricity production and hydrogen production are coupled through electrolysis-based hydrogen generation and hydrogen-based electricity generation. Both electricity and hydrogen are coupled to the CO_2_ supply chain because both sectors can deploy CCS technologies, including natural gas and biomass technologies. In addition, biomass can be consumed in the electricity or hydrogen production sectors.

The model spatially resolves the availability of resources including VRE, CO_2_ storage, and biomass feedstocks. Produced electricity, hydrogen, and CO_2_ can be moved between regions using dedicated transmission. Existing electricity transmission is represented, while new electricity transmission, as well as new hydrogen and CO_2_ pipelines, can be built when it is economic to do so. Finally, modeling system operations hourly over many representative weeks enables a granular representation of operational constraints as well as energy storage (in both electricity and hydrogen production sectors), which may affect technology deployment choices.

Key constraints incorporated in the MACRO model include: (a) regional supply and demand balances for electricity and hydrogen enforced hourly, (b) hourly capacity factor limits for wind and solar resources in all regions, (c) a planning reserve margin constraint that ensures that the supply of firm capacity exceeds load by a certain amount (see Table S22), (d) spatially resolved limits on CO_2_ storage and biomass availability, (e) operational inventory balances for energy storage at hourly and weekly time-scales, as described elsewhere,^31,32^ (f) regional transmission and network constraints for electricity, hydrogen, and captured CO_2_, (g) ramping limits and minimum and maximum output constraints for power and hydrogen production technologies with limited operational flexibility (Table S23), and (h) policy constraints such as CO_2_ emissions limits. The model and associated documentation is available in a public Github repository.^29^

### 2.2 Model Configuration and Assumptions

We used the nine-region contiguous United States version of MACRO shown in Figure 1C to evaluate net-zero emission scenarios of the coupled electricity-hydrogen production system in 2050. Except for existing electricity generation and transmission, we modeled a greenfield energy system in which hydrogen, CO_2_ management, and bioenergy infrastructures were built from scratch. Annual system operations are approximated by modeling hourly operations over 11 representative weeks. Eight representative weeks were sampled from seven years of hourly VRE capacity factors and a single year of energy demand using a *k*-means clustering based time-domain reduction approach. In addition, three “extreme” weeks representing peak power demand as well as minimum solar and wind availability were also incorporated to account for variability in VRE and energy demands that are not captured by the representative weeks. To approximate annual operations, the sampled weeks were stacked based on their chronological occurrence and weighted based on the number of real weeks associated with each representative week. Further details of the clustering approach can be found in a prior publication^33^ and the Github repository.^29^

The use of representative weeks to approximate system operations may overlook the flexibility of storage resources to shift energy over longer time periods.^31,32^ However, our model representation of energy storage explicitly allows for carry over (positive) between representative weeks through a previously developed formulation,^33^ which tracks storage state of charge: (a) within representative weeks at an hourly resolution and (b) across weeks of the year in aggregate. To test the impact of the representative weeks approximation on model outcomes regarding investment and generation, we compare the model results using 11 representative weeks of system operation versus one year of operations at an hourly resolution. The results, summarized in Figure S17, suggest that use of representative weeks provides a reasonable approximation of the results obtainable from annual operations at an hourly resolution, while reducing computational time for each run by more than an order of magnitude (e.g., 500–600 seconds versus 10,000–13,000 seconds).

Regional hourly electricity demands were sourced from the high electrification with moderate technology advancement scenario from the 2018 Electrification Futures Study.^34^ Regional annual hydrogen demands were taken from the high electrification (E+) demand scenario from the Net-Zero America study^13^ (Table S24). In the model, hydrogen demand for each region is represented as a constant hourly demand such that the sum of hourly demands equals the annual demand. Although the regional distribution of demand reflects assumptions made in the underlying literature source, we do not expect this distribution to substantially alter the key drivers of technology choice discussed in this study.

Initial power generation capacity, as well as operational costs of existing capacity, were obtained from PowerGenome,^35^ which utilizes data from the Energy Information Administration (EIA), National Renewable Energy Laboratory (NREL), and the Public Utility Data Liberation (PUDL) Project as displayed in Table S19. Initial power transmission capacity between regions is obtained from the U.S. Environmental Protection Agency (EPA) version of the Integrated Planning Model (IPM) as shown in Table S9.^30^ Investment and operational cost assumptions for greenfield power sector technologies are from NREL’s Annual Technology Baseline (ATB) 2021 and shown in Tables S10 and S12.^36^ Regional investment cost multipliers and fuel costs were obtained from EIA’s Annual Energy Outlook (AEO) 2021 as shown in Tables S20 and S21, respectively, to reflect regional variations in costs.^37^ Hourly VRE profiles were quantified using the ZEPHYR (Zero-emissions Electricity system Planning with HourlY operational Resolution) model, in which solar and wind site capacities and variability profiles were generated using NREL databases such as NSRDB (National Solar Radiation Database), WTK (WIND Toolkit), and the ReEDS (Regional Energy Deployment System) model.^38−41^ A summary of regional wind and solar profiles, as well as the maximum VRE capacity allowed on land areas can be found in Table S25.

In the hydrogen production sector, cost and performance assumptions for electrolytic H_2_ were taken from the International Energy Agency (IEA)’s Future of Hydrogen study,^42^ while assumptions for blue H_2_ were taken from a TEA study by the National Energy Technology Laboratory (NETL),^6^ and H_2_ storage assumptions from Papadias and Ahluwalia,^43^ as shown in Tables S11 and S13. Hydrogen storage costs are based on compressed gas storage in pipes and include the cost of H_2_ compression and storage.^43^ Assumptions for 2050 electrolyzer investment costs are consistent with costs derived used learning curves assuming hydrogen deployment comparable to other net-zero by 2050 studies.^44,45^ We also consider a sensitivity case with higher electrolyzer investment costs based on IEA in the Supporting Information (SI).^42^ Cost assumptions for hydrogen pipelines were taken from Hydrogen Delivery Scenario Analysis Model (HDSAM)^46^ as shown in Table S17.

We further assume that (a) electrolyzers can operate flexibly, with a minimum output level of 10% to minimize concerns about accelerated degradation due to startup and shutdown;^47,48^ (b) blue H_2_ facilities operate with comparatively less flexibility due to the high-temperature, thermo-catalytic nature of the process (near 1000 °C), with higher minimum operating levels of 85% and 50% in the low and high blue H_2_ production flexibility scenarios, respectively; (c) hydrogen-based power technologies are modeled as combined cycle (CC) and combustion turbine (CT) power plants utilizing hydrogen as fuel, with costs and heat rates based on similar natural gas-based generation resources from ATB 2021 shown in Table S10;^36^ and (d) hydrogen transmission between regions via pipelines is modeled by sizing pipeline capacity assuming separate pipeline capacity for flow in each direction. Hydrogen and power transmission within a region are assumed to occur without additional cost, although the amount of transmission needed would depend on the extent to which production and load could be co-located.

In the CO_2_ supply chain, input assumptions for DAC technologies were obtained from the literature as summarized in Tables S14 and S15.^49,50^ Both sorbent and solvent DAC coupled with natural gas generators (with CCS) to provide self-sufficient energy, as well as sorbent DAC powered by externally supplied electricity are represented. The cost and performance assumptions for BECCS electricity and BECCS H_2_ plants were obtained from the Net-Zero America study^13^ as shown in Table S16. Annual CO_2_ sequestration capacities and injection costs were obtained from the ReEDS model, with the former estimated as the annual rate that would attain the same cumulative amount of CO_2_ injection over 100 years^38^ as shown in Table S26. CO_2_ pipeline cost and performance assumptions are provided in Table S18.^51^ Regarding biomass supply, the 2016 Billion-Ton Report was used to quantify biomass availability and cost as summarized in Table S27.^52^ Cost data in this study has been converted to 2019 USD to standardize differences in dollar-years between sources.

### 2.3 Scenarios

Figure 1B shows the core scenario matrix for this study, spanning alternative assumptions for NETs (horizontal axis) and H_2_ infrastructure availability and flexibility (vertical axis), with all scenarios run under a constraint of net-zero CO_2_ emissions in 2050. Along the horizontal axis, scenarios labeled “DAC” allow DAC as the only negative emissions technology, whereas scenarios labeled “All NETs” allow DAC in addition to BECCS in electricity generation and BECCS in hydrogen production. In effect, the “All NETs” scenarios assume availability of lower cost CO_2_ removal than the corresponding “DAC” scenarios, allowing us to examine the sensitivity of results to CO_2_ removal costs. Along the vertical axis, scenarios labeled “Full H_2_ Infra” allow hydrogen storage and hydrogen pipelines, whereas scenarios labeled “No H_2_ Infra” do not allow either hydrogen storage or hydrogen pipelines. In addition, scenarios labeled “Hi Blue Flex” allow blue H_2_ to operate between 50% and 90% utilization of installed capacity in any given hour, whereas scenarios labeled “Lo Blue Flex” allow blue H_2_ to operate only between 85% and 90% utilization of installed capacity in any given hour. In subsequent sensitivity analyses, we investigated the impact of increasing flexibility of blue H_2_ above the levels modeled under the “Hi Blue Flex” scenario (see Figure S14). The findings from this analysis illustrate the value of flexibility under different conditions and could inform design of next generation H_2_ systems.

We separately investigate these dimensions under the assumption that the hydrogen production system is “islanded”, meaning that it is not connected to the electricity grid. Other sensitivity cases are mentioned in Section 3 and shown in the SI (Figures S14–S16).

## 3 Results

### 3.1 Electricity and Hydrogen Production at the National Level

**Figure 2:**
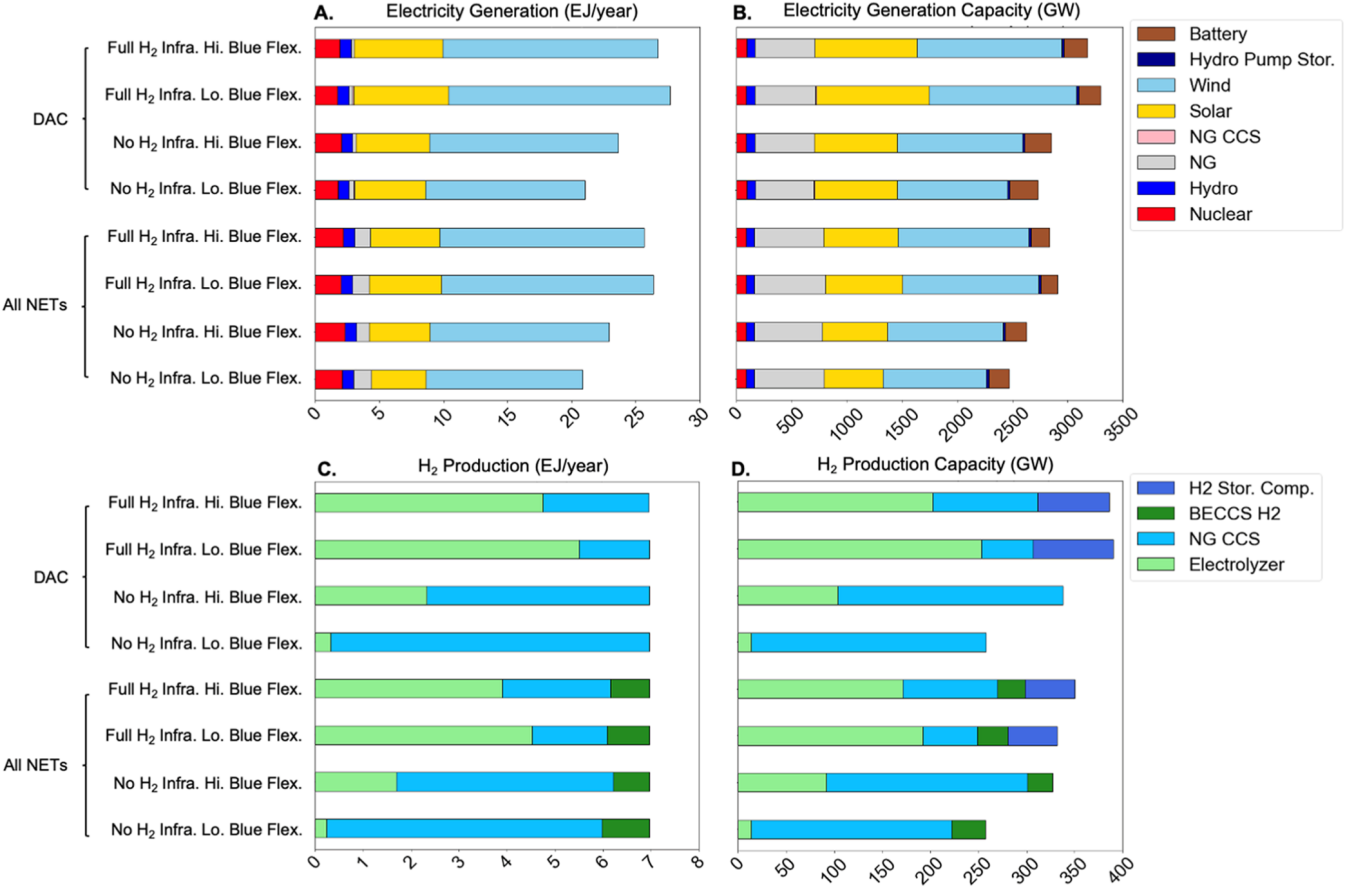
U.S. Electricity generation (A) and hydrogen production (C) and capacity (B,D) across scenarios in 2050 with different assumptions for hydrogen infrastructure flexibility, blue H_2_ operational flexibility, and negative emissions technologies, as described in Figure 1B. CO_2_ capture and sequestration (CCS), bioenergy with CO_2_ capture and sequestration (BECCS), Natural Gas (NG), Storage (Stor.), Compressor (Comp.). Existing power generation capacity modeled for 2050 is available in Table S19. For context, U.S. annual electricity generation and installed electricity generation capacity in 2021 was 4243 TWh and 1,160 GW, respectively.

Figure 2 highlights the impact of hydrogen infrastructure and NETs assumptions on electricity and hydrogen production (Panels A and C) and capacity (Panels B and D). While overall electricity demand increases by 33–43% in the cases with hydrogen storage and pipeline infrastructure that enable electrolytic H_2_, 79–89% of total electricity generation comes from VRE across all cases. In comparison, unabated natural gas accounts for 1–7% of total electricity generation, while unabated natural gas capacity accounts for 18–28% of total electricity generation capacity (528–642 GW) across the cases. The differences between the natural gas share of electricity generation and capacity indicate that unabated natural gas capacity dispatches infrequently, recovering costs by dispatching during times when electricity prices are higher and by contributing to the system planning reserve margin constraint (see Table S22).

Compared to a 100% VRE grid that would require significant investment in other forms of flexibility, deployment of unabated natural gas is a more cost-effective mechanism to provide flexibility when NETs are available to offset remaining emissions.^53−56^ In addition, unabated natural gas is more cost-effective than natural gas CCS in the power sector, because at low capacity factors (1–7%), the emissions penalty of unabated natural gas is lower than the capital cost premium associated with CCS, so natural gas CCS does not deploy in the power sector in these scenarios. Similarly, in these scenarios, hydrogen is not utilized for power generation, because the cost of producing hydrogen is greater than the cost of natural gas, even after accounting for the additional cost of offsetting emissions from natural gas using NETs.

When all NETs are allowed, BECCS H_2_ is preferentially deployed relative to DAC due to its lower cost of removing CO_2_, resulting in lower marginal abatement costs (Table S1). The lower marginal abatement cost effectively lowers the cost of operating emitting generation, which explains why unabated natural gas is 4–7% of generation in the All NETs scenarios but 1–2% of generation in the DAC only scenarios (see emissions balance in Figure S1). Among DAC technologies, solvent-based DAC with onsite gas-based power generation with CCS is selected due to the lower assumed costs compared to sorbent-based DAC powered by onsite gas power generation or grid electricity (Table S14).

Deployment of BECCS in the hydrogen production sector rather than in the electricity generation sector reflects the lower cost and higher energy efficiency of the former (see Table S16 for the technology cost and performance assumptions). Deploying BECCS H_2_ can displace some blue H_2_ or electrolytic H_2_, depending on which would otherwise be the marginal production technology. However, BECCS H_2_ is typically only deployed in a few regions since the amount of CO_2_ removal is relatively small and some regions may have more favorable conditions for BECCS deployment than others (Table S27). Since the amount of CO_2_ removal required to offset remaining emissions is modest in these scenarios, biomass use is small relative to the available resource.

Across the cases, we find that the mix of hydrogen production is primarily affected by assumptions about the availability of hydrogen storage and pipeline infrastructure, and less so by assumptions about blue H_2_ operational flexibility or NETs availability. With all hydrogen infrastructure available, electrolytic H_2_ accounts for the largest share of production (68–79% in the DAC only scenarios and 56–65% in the All NETs scenarios). In contrast, when hydrogen storage and pipeline infrastructure is assumed to be unavailable, blue H_2_ accounts for the largest share of hydrogen production (67–95% in the DAC only scenarios and 65–82% in the All NETs scenarios).

The role of hydrogen infrastructure availability in driving differences in electrolytic and blue H_2_ production can be explained by the differences in annual average electricity prices (see eq S1 in SI Section 7) and those paid by the electrolyzer (assumed to pay during hours when electricity is consumed as shown in eq S2 in SI Section 7). For instance, consider the DAC only scenario with hydrogen infrastructure availability and low blue H_2_ production flexibility, in which, for the NCEN region, the average electricity price is $42 per MWh (see Table S3), while the electricity price weighted by hourly electrolyzer power consumption is $31 per MWh (see Table S3, with results for other scenarios in Tables S4–S6). For context, an electricity price of $33 per MWh equalizes the levelized cost of electrolytic H_2_ and blue H_2_ production at around $1.8 per kg for the technology assumptions used and capacity factors based on model outputs (see Figure S5). This cost does not include the cost of delivering hydrogen to end users. Without hydrogen storage and pipelines, the electrolyzer would have to operate continuously to meet demand in all hours, which would raise the average price it pays for electricity above the $33 per MWh breakeven price, thereby rendering it more expensive than blue H_2_. Related to this, lower capital costs for VRE and battery storage reduce electricity prices overall and thus lead to greater electrolyzer deployment (see Figures S14 and S15), while the opposite is true with increased VRE and battery storage capital costs.

The CO_2_ emissions cost associated with blue H_2_, using the marginal CO_2_ abatement cost (the shadow value on the system-wide CO_2_ emissions constraint), is relatively small (∼$0.1 per kg in the All NETs case and $0.3 per kg in the DAC case as shown in Table S1) due to the high assumed CO_2_ capture rate. In addition, the added cost of hydrogen storage for electrolytic H_2_ production, when levelized over total electrolytic H_2_ generation, is $0.06–$0.17 per kg across the regions in the DAC only scenario with full hydrogen infrastructure and low blue H_2_ production flexibility. This cost is low relative to the cost of electrolytic H_2_ production because storage is operated frequently, with approximately 50–200 cycles per year. Here, cycles are defined as the ratio of annual energy withdrawn from storage to the installed energy storage capacity. For context, the corresponding range for Li-ion battery storage for the full H_2_ infrastructure + low blue flex + DAC scenario is 116–256 cycles per year.

Increasing blue H_2_ flexibility has a larger effect on technology selection when hydrogen storage and pipeline infrastructure are unavailable, leading to an increase in the electrolytic H_2_ share from 5% to 33% in the DAC only scenarios and from 3% to 24% in the All NETs scenarios. To some extent, blue H_2_ operational flexibility substitutes for the lack of other forms of flexibility, allowing electrolytic H_2_ to minimize production during hours with the highest electricity prices. Indeed, when blue H_2_ operational flexibility is assumed to be close to electrolytic H_2_ operational flexibility (allowed to vary between 10% and 90% of nameplate capacity), the electrolytic share increases to 63% (Figure S14), similar to the share attained when hydrogen storage and pipelines provide flexibility. Interestingly, while improving the performance of a technology would typically increase its deployment, we find that increasing the flexibility of blue H_2_ could lower the amount of production from blue H_2_, with the additional flexibility of blue H_2_ enabling more electrolytic H_2_ in the absence of hydrogen storage and pipelines.

When hydrogen storage and pipelines are available, the impact of increasing blue H_2_ operational flexibility is relatively small. Since the flexibility from hydrogen storage and pipelines allows electrolytic H_2_ to avoid the hours with the highest electricity prices, the benefit from additional flexibility provided by blue H_2_ is limited. This is substantiated by the similar levels of blue H_2_ deployment observed in the low blue H_2_ production flexibility case and a sensitivity case in which blue H_2_ is assumed to have flexibility close to electrolytic H_2_ (see Figures S14 and S15).

Overall, we find that flexibility in any form allows the hydrogen production system to dispatch electrolyzers at times when their production cost would be lower than that associated with blue H_2_. Since hydrogen prices reflect the cost of the marginal production technology, hydrogen prices are effectively capped by the cost of blue H_2_ production. Average prices are then modestly lower when electrolytic hydrogen accounts for a greater share of total production (see Table S1).

### 3.2 Regional Results

**Figure 3:**
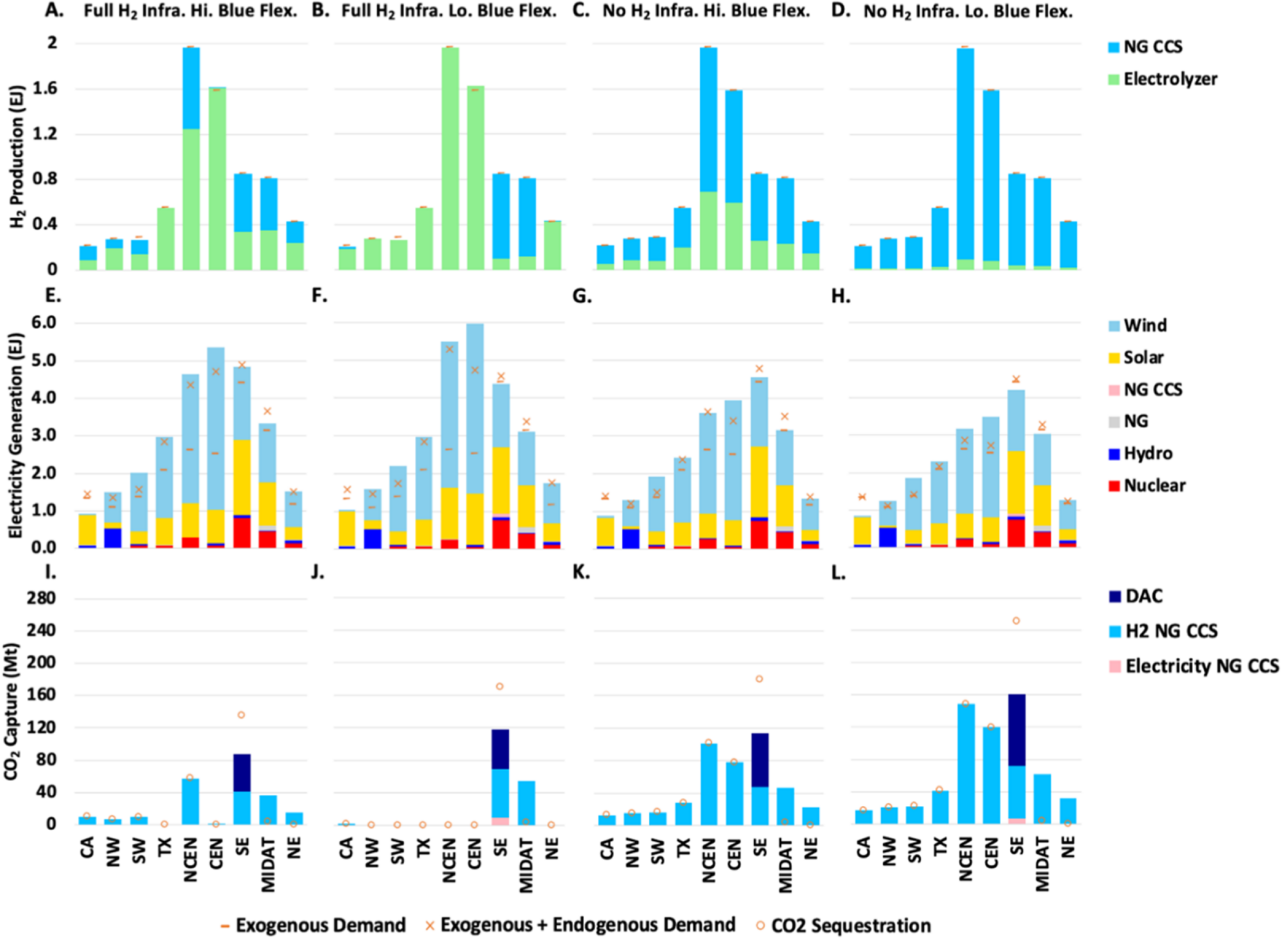
Regional electricity generation, hydrogen production, and CO_2_ captured by technology with different assumptions about hydrogen infrastructure flexibility and blue H_2_ operational flexibility in 2050. Results are shown for the DAC only scenarios, i.e., DAC is the only NET. The orange line on each bar indicates exogenous demand (for electricity or hydrogen) in each region, and the orange “*x*” indicates the total (exogenous plus endogenous) electricity demand in each region (there is no endogenous demand for H_2_ in these cases). If the bars are lower than the orange markers in a given region, then that region is a net electricity or hydrogen importer, and vice versa. There are losses associated with electricity transmission and storage, which implies that total generation will exceed demand. Orange circles in the bottom panel correspond to CO_2_ sequestered in each region. When CO_2_ sequestered exceeds CO_2_ captured in a given region, then that region is a net importer of captured CO_2_, and vice versa. The regional acronyms are defined and their geographic locations are shown in Figure 1C. Similar results for the All NETs scenarios are shown in Figure S7. Capacity results for the DAC and All NETs scenarios are shown in Figures S6 and S8, respectively.

The regional mixes of electricity and hydrogen production and capacity (Figures 3 and S6 respectively) are broadly similar to the national results in Figure 2 with some minor exceptions. When blue H_2_ operational flexibility is low, each region produces mostly electrolytic or mostly blue H_2_ depending on the availability of hydrogen storage and pipeline infrastructure. For instance, under full H_2_ infrastructure availability (Full H_2_ Infra., Lo Blue Flex), two regions deploy mostly blue H_2_ (SE and MIDAT), even though electrolytic H_2_ dominates in other regions and therefore at the national level. These regions have higher average electricity prices ($46–49 per MWh) and lower average delivered natural gas prices ($3.7/MMBtu) than the national average (Tables S3 and S21), which lowers the break-even electricity price below which electrolytic H_2_ would be selected (around $30 per MWh versus $33 per MWh mentioned earlier). When blue H_2_ operational flexibility is high, there is more mixing of blue and electrolytic H_2_ at the regional level, which is most notable in the case without H_2_ storage and pipeline infrastructure. As discussed earlier, this result occurs because blue H_2_ provides some additional flexibility for electrolytic H_2_.

Across the scenarios, hydrogen production is generally co-located with demand, indicated by the relatively small differences between supply (top of each bar) and demand (marker indicating exogenous demand). This is generally not the case in the power sector, as many regions buy or sell to neighboring regions, indicated again by the differences between the top of each bar and the marker showing total demand.

Incremental electricity transmission expansion can be avoided altogether when electricity is produced in the same region as the hydrogen production that consumes it. Figure S9 shows that incremental electricity production is typically similar to electricity demand for electrolytic H_2_ in regions with the most electrolytic H_2_ production. This implies that most of the electricity supply for electrolytic H_2_ production in these regions can be met using local VRE resources (Figure 3).

The regional distribution of blue H_2_ production is the primary driver for CO_2_ pipeline expansion. Given the relatively high cost of hydrogen transmission, it is more cost-effective to deploy blue H_2_ to meet local hydrogen demand and to transport the captured CO_2_ out of regions with limited geological CO_2_ storage capacity such as the Northeast and Mid-Atlantic. To quantify this point, the CO_2_ pipeline investment cost (on per MtCO_2_-mile basis, shown in Table S18) can be converted into energy throughput units ($/MW H_2_-mile) by multiplying by CO_2_ captured per tonne of H_2_ produced from blue H_2_ plants (around 9.5 tCO_2_/tH_2_). This yields an effective CO_2_ pipeline investment cost of 661 $/MW H_2_-mile which is much lower than the investment cost of H_2_ pipelines. As such, CO_2_ network expansion is greater when the blue H_2_ share is higher (e.g., Figure 3L vs 3K). In contrast, the availability of NETs does not affect the magnitude of CO_2_ pipeline investment (see Figure S4), as NETs are mainly deployed in regions with low NG prices (which is used to provide energy inputs for deployed DAC technology), relatively low CO_2_ storage costs, and high CO_2_ storage potential that do not require CO_2_ to be exported for storage (e.g., SE region in Figure 3).

### 3.3 Electricity and Hydrogen System Operation

**Figure 4:**
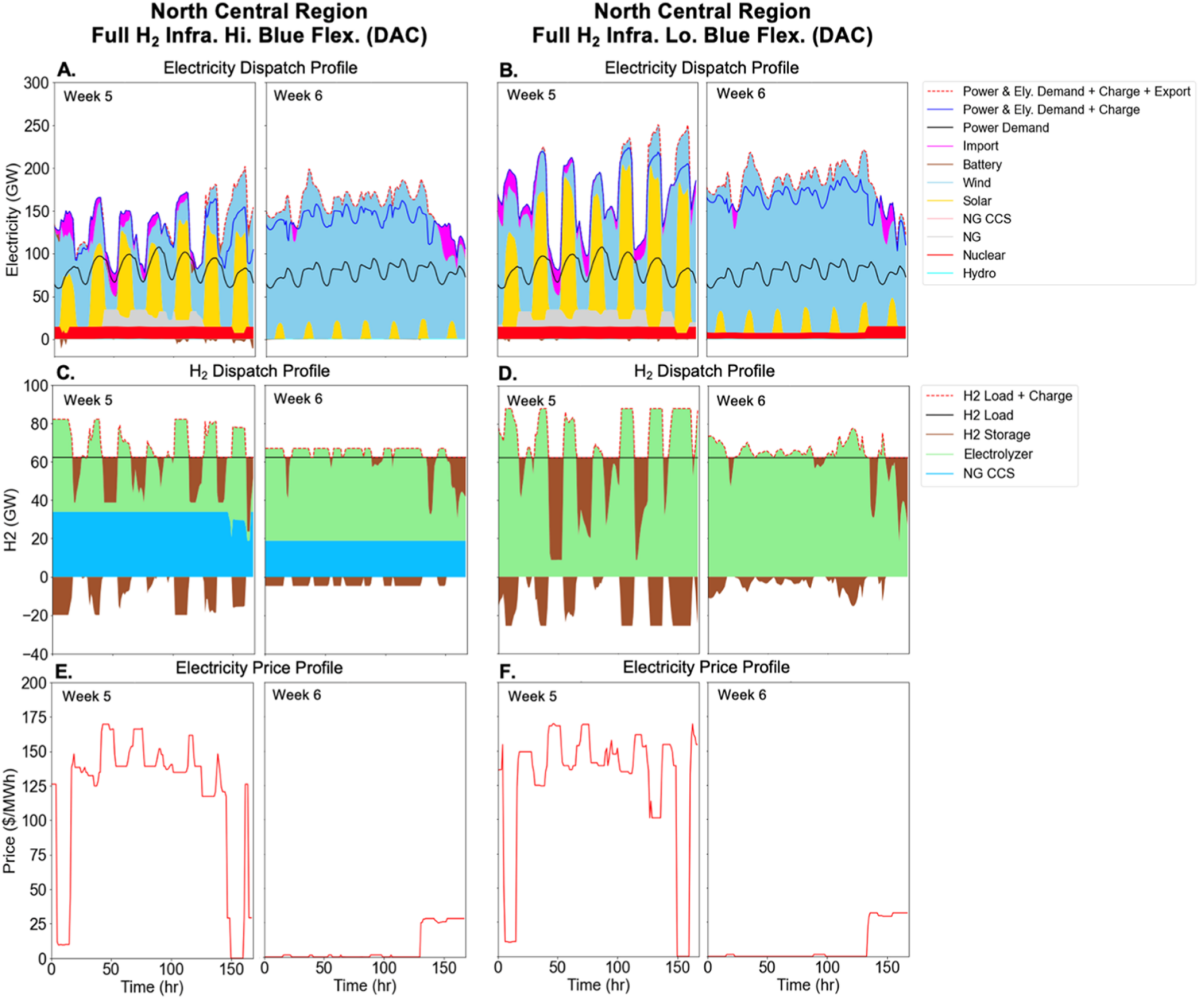
System operation for electricity (Panel A, B) and hydrogen (Panel C, D) production sectors, as well as electricity prices (Panel E, F) for two representative weeks in the north central (NCEN) region. DAC only scenarios with full hydrogen infrastructure are shown, with high blue H_2_ flexibility in panels A, C, E, and low blue H_2_ flexibility in panels B, D, F. The left half of each panel shows a representative week in summer with limited wind but abundant solar resources, whereas the right half of each panel shows a representative week in winter when the wind resource is abundant but solar is limited. Panel E and F plot the energy component of electricity prices (left *y*-axis) using the dual variable on the hourly supply demand balance constraint. Similar results for the All NETs scenarios are shown in Figure S10. Electrolyzer (Ely).

**Figure 5:**
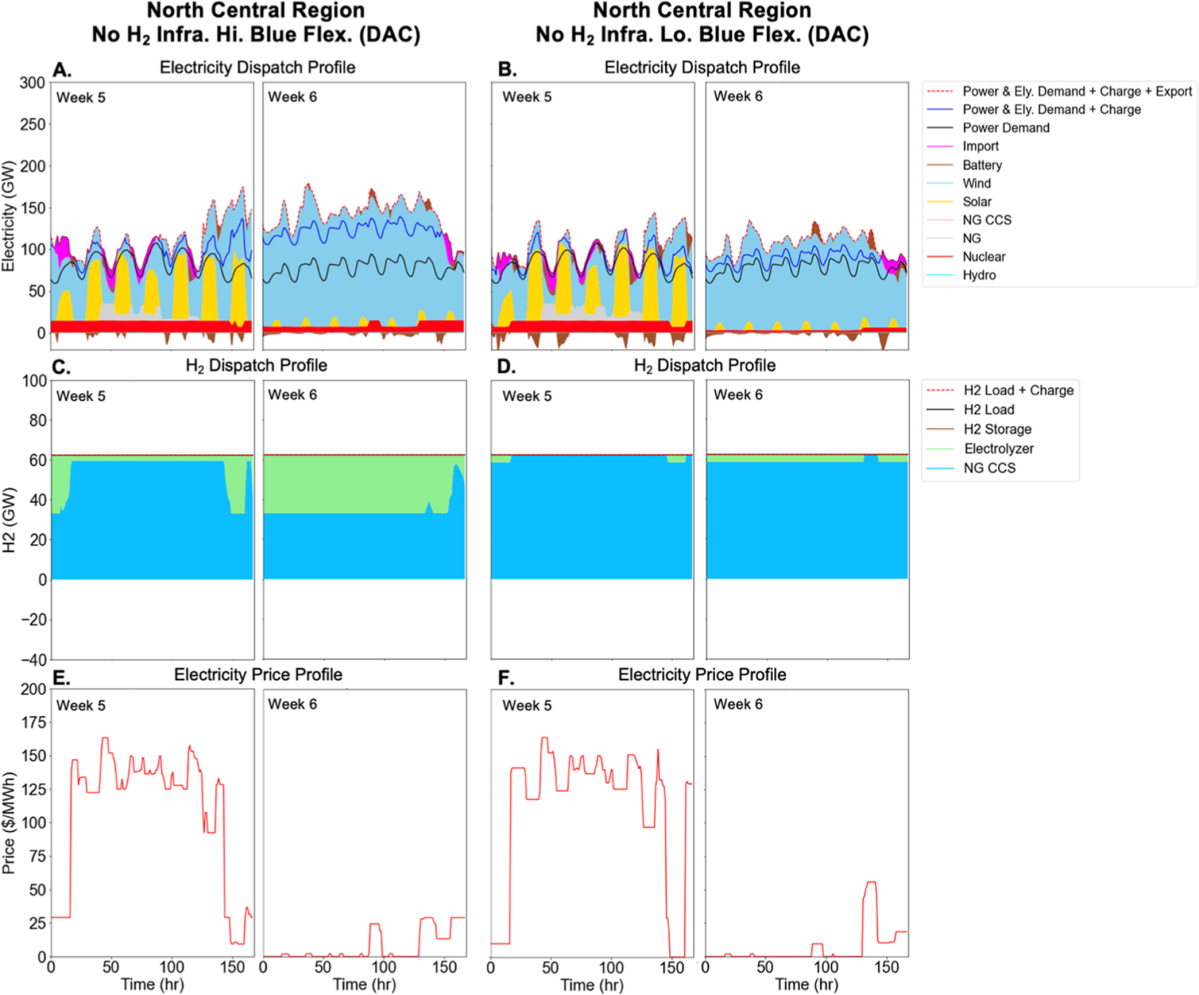
Dispatch profiles for electricity (Panel A, B) and hydrogen (Panel C, D) production sectors, as well as electricity prices (Panel E, F) for two representative weeks in the north central (NCEN) region. DAC only scenarios with no hydrogen storage and pipeline infrastructure are shown, with high blue H_2_ flexibility in panels A, C, E, and low blue H_2_ flexibility in panels B, D, F. The left half of each panel shows a representative week in summer with limited wind but abundant solar resources, whereas the right half of each panel shows a representative week in winter when the wind resource is abundant but solar is limited. Panel E and F plot the energy component of electricity prices (left *y*-axis) using the dual variable on the hourly supply demand balance constraint. Similar results for the All NETs scenarios are shown in Figure S11. Electrolyzer (Ely).

To further understand the roles of electrolytic and blue H_2_, we analyze the hourly electricity and hydrogen dispatch of two selected representative weeks (one in summer and one in winter) in the north central region (NCEN), which has the greatest assumed hydrogen demand in the study,^13^ under full (Figure 4) and no H_2_ infrastructure assumptions (Figure 5).

In general, electrolyzers operate less during times of high electricity prices (when VRE generation is lower and electricity end use demand is higher) and more during times of low electricity prices (when VRE generation is higher and end use demand is lower). In the scenarios with full H_2_ infrastructure (Figure 4), low VRE availability periods coincide with the periods of lowest electrolyzer operation and greatest hydrogen storage discharge (Figure 4C,D). When allowed to operate with higher flexibility (between 50% and 90% capacity factor; left panels), blue H_2_ operates at higher capacity factors when electricity is expensive, reducing the need to run electrolyzers at this time. However, due to the minimum output limit, it also must run to some extent at other times, partially foregoing the opportunity to run electrolyzers during times of lower electricity prices. Therefore, the optimal level of blue H_2_ deployment is largely determined by balancing the benefits (avoiding electrolytic H_2_ production during times of higher electricity prices) and costs (foregone benefits when electrolyzers cannot be run fully at times of low electricity prices). When the operational flexibility of blue H_2_ is constrained to operate between 85% and 90% capacity factor (Figure 4B,D,F), it is more cost-effective to deploy electrolytic H_2_ exclusively, as the foregone benefits associated with not running electrolyzers are higher when blue H_2_ is forced to run more.

Because there is more electrolytic H_2_ in the case with low blue H_2_ flexibility and because there is no blue H_2_ to buffer electrolyzers from higher electricity prices, there is a need for more hydrogen storage in this case. In effect, for a given amount of hydrogen production, the operating profile of electrolyzers reflects a balance between the incremental cost of hydrogen storage and the incremental cost of purchased electricity. The potential for higher realized electricity prices when electrolyzers must meet demand in all hours justifies greater hydrogen storage investment. With more storage, the average capacity factors of electrolyzers drop from 74 (left) to 69% (right), as shown in Table S2.

While the power dispatch patterns are similar in the scenarios without hydrogen storage and pipeline infrastructure (Figure 5A,B), the total power demand decreases significantly with the relatively low electrolytic share of hydrogen production (Figure 5C,D). When blue H_2_ is allowed to operate with high flexibility (Figure 5C), blue H_2_ ramps down to its minimum capacity factor (50%) during periods of low electricity prices, allowing a greater overall share of electrolytic H_2_. During the periods with expensive electricity, electrolyzers operate at their minimum capacity factor (10%) as blue H_2_ operates at its maximum (90%) capacity factor. On the other hand, when blue H_2_ operational flexibility is low (Figure 5D), electrolyzer deployment is minimized to avoid periods of expensive electricity prices. Given the narrow range of blue H_2_ operation (85–90% capacity factor), any electrolyzer operation during periods of low electricity prices would also be forced to operate at periods of high prices due to the absence of flexibility.

Without hydrogen storage and pipeline infrastructure, there is more battery storage charging and discharging than in the cases with hydrogen storage and pipeline infrastructure (compare Figures 4A,4B and 5A,5B). With full H_2_ infrastructure availability, electrolyzers can add demand-side flexibility to the electricity system, which can partially substitute for battery storage. This flexibility facilitates not only the integration of incremental VRE expansion for electrolyzers, but also integration of VRE used to meet exogenous electricity demand. As a result, battery storage investment is substantially lower in the more flexible cases even though there is more overall VRE deployment (see Figure 2B).

### 3.4 Grid-Connected versus Islanded Electrolytic H_2_ Production

While the cases above examine the role of grid-connected electrolytic H_2_, there is growing interest in “islanded” electrolytic H_2_ production systems in which dedicated VRE generation and energy storage power a co-located electrolyzer.^57^ Such a system could avoid interconnecting to the grid and would inherently produce low-carbon hydrogen. At the same time, the entire cost of electricity generation in an islanded system must be borne by hydrogen producers, since the investment and operation of electricity generation and storage in an islanded system solely reflect electricity demand associated with hydrogen production.

For modeling purposes, the islanded system is differentiated from the grid-connected system in three ways. First, there is no exogenous power demand and no existing power generation, storage or transmission capacity in the islanded system. Instead, a greenfield electricity system serves demand from electrolyzers. Second, only VRE and battery storage capacity can be built in this greenfield electricity system, meaning that there is no natural gas electricity generation or electric transmission capacity to provide additional flexibility. Third, there is no planning reserve margin constraint (see Section S9) in the greenfield electricity system, consistent with how such systems are typically defined. All H_2_ demand in these scenarios is served by either islanded green H_2_ or blue H_2_.

**Figure 6:**
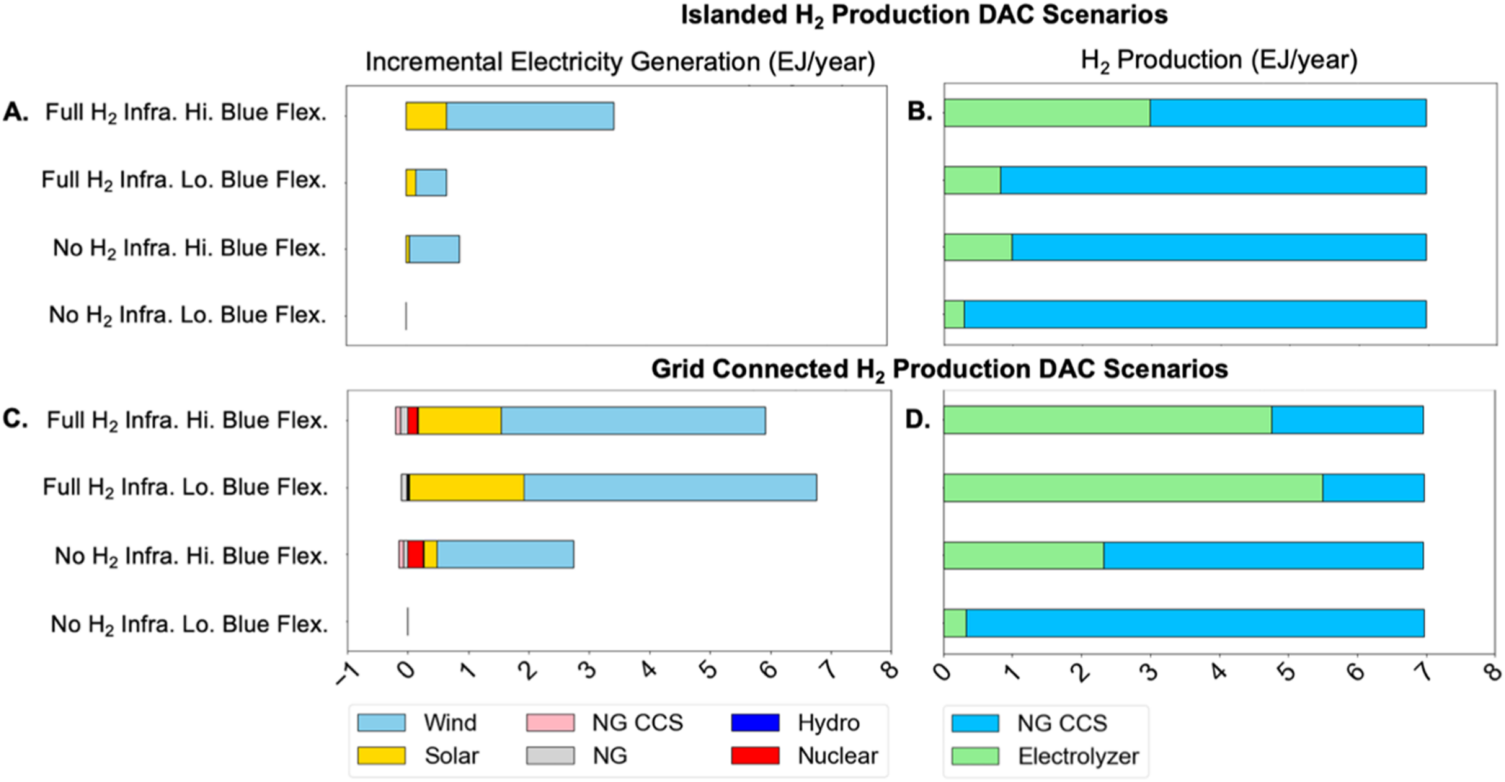
Incremental electricity generation (A,C) compared to the least flexible hydrogen infrastructure scenario (no H_2_ infrastructure + Lo Blue Flex.) and absolute hydrogen production (B,D) for DAC only scenarios under islanded and grid-connected systems for the contiguous U.S. in 2050. The islanded system (panels A,B) is differentiated from the grid-connected system (panels C,D) in the following ways: (a) no exogenous power demand and no existing power generation, storage or transmission capacity, (b) only VRE and battery storage capacity can be added in the power sector, and (c) no planning reserve margin constraint (see Table S22). Grid-connected hydrogen production scenarios are identical to those in Figure 2.

Figure 6 shows deployment of electrolytic and blue H_2_ in an islanded system compared to deployment in the corresponding grid-connected systems discussed in Figures 2–5. As in the grid-connected cases, increasing electrolyzer flexibility by allowing hydrogen storage infrastructure increases the share of electrolytic H_2_ production. In addition, increasing blue H_2_ flexibility also increases the electrolytic H_2_ share, with or without hydrogen storage infrastructure. However, for the same hydrogen infrastructure availability assumptions, the share of electrolytic H_2_ production is substantially lower in the islanded system than in the grid-connected system, with the largest differences observed in the cases with hydrogen storage and pipeline infrastructure. For example, the electrolytic H_2_ share in the case with hydrogen storage and pipeline infrastructure and low blue H_2_ production flexibility is 79% and 11% in the grid-connected and islanded systems, respectively.

As highlighted in Figure 4C and 4D, electrolyzer operational flexibility in the grid-connected system allows electrolyzers to increase production during times of high VRE output and to reduce production during periods of low VRE availability. So, in a grid-connected system, the incremental VRE capacity added to serve electrolyzer power demand during high VRE availability periods can also serve other electricity loads during times of lower VRE output when electrolyzer output is minimized. For example, if solar PV operates at different capacity factors in two adjacent periods, with excess production relative to load in the first period, then battery storage could move electricity across periods. If an additional incremental of PV is added to serve demand from electrolyzers in the first period, then that PV can serve the electricity load in the second period (assuming the electrolyzer can turn down in the second period), reducing the need to discharge batteries. As a result, there is less need for battery storage to shift generation from periods of higher VRE output to periods of lower VRE output.

In Figure S12, which shows incremental electricity capacity and hydrogen capacity in the same cases, we observe a decrease in incremental battery storage capacity in the grid-connected system, whereas there is little change in battery storage capacity in the islanded system. On the other hand, hydrogen storage capacity increases more in the grid-connected system than in the islanded system (Figure S13). Because some of the incremental VRE in the grid-connected system is used to serve other loads, the VRE used for electrolysis is effectively more variable from the perspective of the electrolyzer, resulting in lower electrolyzer capacity factors (see Table S7). This variability is managed with additional hydrogen storage.

Put differently, there is a trade-off between incremental battery storage investment and hydrogen storage investment. Because battery storage is costly relative to hydrogen storage on a $ per kWh of energy storage capacity basis ($113/kWh for battery storage–see Table S10 versus 17 $/kWh_H2_ for hydrogen storage–see Table S11), it is cost-effective to add hydrogen storage when it allows battery storage investment to be avoided, which occurs in the grid-connected system. Such a system is more favorable to electrolyzer deployment because the effective cost of purchased electricity is lower, reflecting a more efficient use of resources in the electricity sector.

When electrolyzer flexibility is minimized, such as in the case without hydrogen storage infrastructure and low blue H_2_ flexibility, the shares of electrolytic production in islanded and grid-connected systems are similar (Figure 6C,D). However, even a small increase in electrolyzer flexibility enabled by increased blue H_2_ flexibility leads to more electrolytic production in the grid-connected system than in the islanded system. This further supports the finding that utilizing electrolyzer flexibility for grid balancing reduces electricity costs for hydrogen production in grid-connected systems.

Figure 6 highlights that the availability of electrolyzer flexibility in the grid-connected system also changes the incremental generation mix compared to the islanded system, with greater reliance on solar generation in the grid-connected case (See Table S8). As solar generation adoption is often accompanied by Li-ion battery storage due to its diurnal cycle, electrolyzer flexibility can reduce the cost of integrating solar by reducing the need for battery storage, although this increases the need for hydrogen storage (Figure S13). This effectively amounts to reducing the net cost of storage, thereby increasing solar deployment (relative to wind) compared to the islanded case. This result is consistent with previous studies showing that electrolyzer flexibility (and more generally demand flexibility) can limit the rate at which VRE value declines with deployment.^58^

## 4 Discussion

### 4.1 Hydrogen as a Potential Source of Electricity System Flexibility

Flexible electrolyzer operation, enabled in part by hydrogen storage, allows scheduling electricity demand for hydrogen production to align with the hours with lowest electricity prices, which are the hours with high VRE generation.^59,60^ Additionally, the flexibility of electrolyzers displaces other forms of grid-side flexibility such as battery storage to serve the same purpose, thereby lowering the cost of integrating VRE. In this way, coupled electricity-hydrogen systems may lead to higher shares of VRE in the electricity generation mix.^61^ The mutual benefits of electricity and hydrogen system coupling are also apparent in the sensitivity cases (Figures S14 and S15), which show that lowering capital costs of VRE and battery storage increases electrolyzer deployment and VRE shares for the same level of hydrogen infrastructure flexibility, while increasing capital costs of VRE and battery storage have the opposite effect.

This demand-side flexibility is quite different than the other way in which hydrogen could provide flexibility to the electricity system–as dispatchable generation or long-duration energy storage.^62^ Because the cost of producing hydrogen is typically higher than the cost of natural gas, it is generally more cost-effective to use natural gas as firm capacity and as a peaking resource when the cost of offsetting residual emissions (which are small if dispatched infrequently) via NETs is low. Higher assumed costs of DAC or higher natural gas prices could lead to deployment of H_2_ as a peaking resource in power generation. However, based on cost and performance assumptions for NETs and natural gas prices assumed in this study, the cost of deploying natural gas for peaking in power generation is lower than the cost of using H_2_ for the same purpose. As a result, we do not observe any deployment of hydrogen as a source of supply side flexibility to the electricity system in the scenarios considered here. Overall, we conclude that hydrogen may provide more flexibility to the electricity system as a demand-side resource rather than as a supply side resource.

### 4.2 Grid Connection as an Electrolysis Enabler

The differences in electrolyzer deployment between the grid-connected and islanded cases imply that it is more cost-effective to couple electricity and hydrogen production than to produce the same quantities of electricity and hydrogen in separate unconnected systems. Interestingly, we find that the optimal islanded electrolytic H_2_ configuration is not substantially different than the optimal grid-connected electrolytic H_2_ configuration (measured in this case by incremental electricity generation), when normalized for differences in the amount of electrolytic production. Both systems primarily deploy VRE, have similar levels of curtailment (Below 10%, see Table S7), and have similar annual electrolyzer capacity factors (around 70–80% as shown in Table S7).

However, the difference in electricity supply cost between the grid-connected and islanded cases reflect real resource costs borne or avoided by electrolyzers that materially impact their competitiveness relative to blue H_2_. In the islanded cases, the full cost of VRE and electrolyzer investment is borne by electrolyzers, but in the grid-connected cases, the incremental cost is lower. While battery costs are not directly borne by electrolyzers in the grid-connected cases, avoiding battery investment lowers the electricity system resource cost and the electricity prices in hours when electrolyzers are running.

Whether electrolyzers can fully realize the benefits of grid connection depends to some extent on how demand resources are managed within wholesale electricity markets.^63,64^ Currently, wholesale electricity markets across the U.S. and in other regions are primarily designed to optimize supply to satisfy fixed demand at a given time. Demand participation in these markets is limited to programs that incentivize demand reduction during certain grid-stressed periods.^65^ Although the opportunity for flexible demand to bid into day-ahead electricity markets, i.e., be co-optimized with supply based on cost, currently exists in most wholesale electricity markets, there is limited information about the use and practical limitations of this mechanism.^65^ In addition, whether electrolyzers and other flexible loads can avoid firm capacity payments would further affect the benefits of grid connection. These considerations also presume that electrolyzers can participate in wholesale electricity markets and therefore avoid electricity distribution costs. If such benefits of grid connection cannot be realized, or if there are other practical barriers to grid connection, islanded H_2_ systems could become more appealing.

### 4.3 Factors Affecting Electrolyzer Flexibility

Results from this study highlight how electrolyzer flexibility impacts the competitiveness of electrolytic H_2_ versus blue H_2_ in a cost-optimized net-zero system. In addition to the institutional factors discussed above, realizing electrolyzer flexibility in practice is also contingent on several technology-specific factors. First, across the cases analyzed, hydrogen storage is the main lever to enhance electrolyzer flexibility, with installed capacities capable of meeting average hourly hydrogen demand for 100 to 200 hours (Figure S2) in the grid-connected scenarios. For context, in 2022, U.S. natural gas consumption was 32.2 trillion cubic feet (tcf),^66^ and salt cavern storage capacity, representing 15% of total gas storage, was 0.708 tcf,^67^ which corresponds to around 190 hours of average hourly natural gas demand. This suggests that the scale of hydrogen storage being considered has precedent in other parts of the energy system. That said, practical limitations on land area, safety, material requirements, or other factors could limit the deployment of pipe-based hydrogen storage and indirectly limit electrolyzer flexibility. In addition, the economics of storage depend to some extent on how storage is operated, and there could be additional considerations that limit the amount of cycling and implied arbitrage value of storage.

At the same time, while these storage technologies have geographic flexibility, they are an order of magnitude more expensive than geological storage in salt caverns, which is the dominant method of large-scale hydrogen storage today.^43^ Such considerations suggest that regions with access to geological salt cavern hydrogen storage could be particularly advantaged for electrolytic H_2_ production. As an illustration of the impact of low-cost geological H_2_ storage^43^ availability, we modeled a case in which it could deploy in regions with substantial available resources previously identified by other studies^68,69^ (TX, SW, NCEN). Unsurprisingly, this case leads to greater deployment of hydrogen storage and electrolyzers, along with increased hydrogen transmission to access the geographically constrained storage resources (Figure S16).

Second, the feasibility and extent of blue H_2_ operational flexibility, which has a substantial impact on outcomes in cases without hydrogen storage, remains to be demonstrated at scale. An individual blue H_2_ facility today, relying on high operating temperature (near 1000 °C) reactors heated by natural gas combustion, may have limited operational flexibility.^6^ However, the operational flexibility of a fleet of blue H_2_ plants might be achieved by turning on and off individual plants, each with limited operational flexibility, assuming they could be sufficiently coordinated. Even so, incentives to run any blue H_2_ plant at a lower capacity factor depend on a mechanism to effectively enforce merit order dispatch across the hydrogen production system. While a market mechanism could emerge if the scale of hydrogen production were to become sufficiently large, it is also possible that mixed fleets of blue H_2_ plants and electrolyzers could be owned and operated by a single entity with an incentive to operate the broader fleet in the most cost-effective manner. Finally, findings regarding the impact of blue H_2_ flexibility assume no change in the electricity intensity of the blue H_2_ process with increasing flexibility. Recent advancements in methane reforming technology suggest use of electricity-based heating, which could improve operational flexibility but would also increase the electricity intensity of the process^70^ and thus could lead to different outcomes than those observed here.

Third, we adopt a common modeling assumption that electrolyzers have a high degree of operational flexibility, constant energy efficiency, and no flexibility-related operating costs (e.g., accelerated degradation due to cycling). Although electrolyzer flexibility is well-documented, there is limited information about cell durability and accelerated degradation from such operation. Process-level analysis has suggested that incentives to operate electrolyzers flexibly would be reduced if use-dependent degradation were included in operational decisions.^71^ On the other hand, we assume that electrolyzers are assumed to operate at a minimum of 10% of the built capacity in any hour, which somewhat limits the value of flexibility because electrolyzers are never fully shielded from the hours with highest electricity prices. Relaxing this assumption would tend to increase electrolyzer competitiveness, as demonstrated by a sensitivity case in which the minimum capacity factor constraint is removed (Figures S14 and S15). Higher electrolyzer capital costs would also implicitly limit their operational flexibility because of the need to recover costs, which lowers deployment relative to blue H_2_ as seen in the sensitivity analysis (Figures S14 and S15).

Finally, it is common to assume that hydrogen production must satisfy a constant exogenous load. However, some final demands for hydrogen may also be flexible. If a substantial share of hydrogen demand were flexible, supply side flexibility would be less likely to drive the selection of hydrogen production technologies.

### 4.4 Role for Transmission

The scenarios analyzed in this study suggest that hydrogen deployment would not, by itself, drive a significant expansion of either inter-region electricity or hydrogen transmission. These findings imply that it is generally cost-effective to satisfy regional hydrogen demand using hydrogen produced in the same region, regardless of how hydrogen is produced. In addition, for blue H_2_, the lack of CO_2_ storage in a region (e.g., NE) does not appear to impede deployment, due to the relatively low cost of CO_2_ transport versus hydrogen transport assumed here. For electrolytic H_2_, the available VRE supply in most regions is sufficient to meet demand from electrolyzers in those regions without the need for incremental electricity imports (Figure S9). These findings suggest that in the absence of any non-economic barriers to VRE deployment, the proposed spatial-matching requirements associated with the hydrogen production tax credit in the U.S. may be readily satisfied in many regions.^72^ In addition, because many regions are relatively independent from one another in terms of hydrogen production, including electricity inputs, it is unlikely that different assumptions about the patterns of regional demand would change the fundamental insights about technology choice discussed earlier.

### 4.5 Limitations of Levelized Cost of Hydrogen (LCOH) Comparisons

Our study highlights that the economic competition between electrolytic and blue H_2_ depends on the profile of hourly electricity prices that can be revealed by modeling the combined system. However, as illustrated in Figures 4 and 5, the cost-optimal electrolyzer operational pattern depends on particular aspects of the scenario, including the composition of the power system and hydrogen infrastructure availability and flexibility, among other factors. This implies that simplified metrics such as annual average electricity prices, even if sourced from electricity systems modeling, may not reflect the realized price paid by electrolyzers, given their potential ability to dispatch flexibly over the year, and would therefore not be particularly useful in LCOH calculations when comparing the costs of electrolytic H_2_ to other hydrogen production technologies. This is a specific example of a more general problem that arises in any sector when comparing profitability of technologies that may be operated differently such that both costs and value (revenue) vary spatially and temporally.^73^ In addition, as discussed above, depending on how electrolyzers are treated as electricity market participants, some costs faced by other loads could potentially be avoided, which implies that using a delivered electricity price in LCOH calculations may not be appropriate.

### 4.6 Study Limitations

Several limitations of this analysis could be expanded in future work. First, the relatively coarse spatial granularity of this model version does not shed light on specific siting considerations or intra-regional transmission needs. Accounting for these factors would require more spatially resolved modeling and accompanying data sets. Second, our analysis is based on approximating system operations using representative period selection methods that may not fully capture the multi-dimensional time series variability and consequently may bias the model’s investment decisions. Future work could consider using more advanced approaches proposed^74−76^ to capture multi-dimensional variability in multi-vector energy system models. Third, we do not consider the time path of hydrogen deployment between the present and 2050, which implicitly assumes that any near-term path dependency, say due to short-term policy support, will not substantially affect choices in the longer term.^13^ Fourth, our analysis does not account for existing hydrogen demands from the industrial sector or the potential to upgrade or retrofit existing carbon-intensive hydrogen production facilities as part of the transition to a net-zero energy system. Accounting for these factors might impact the overall hydrogen production mix, given that competition between new and existing plants (with the option to retrofit) could be different than competition between new builds. Fifth, we did not model other flexible loads in the system that might arise in the future (e.g., electric vehicle charging), which could impact grid electricity prices and thus indirectly affect the cost of electricity used for electrolytic hydrogen production. Finally, although the focus of this paper is on the competition between blue and electrolytic H_2_ in a net-zero system with exogenous demand, the future final demand for hydrogen remains quite uncertain. By including final demand for liquid fuels and considering competition among fuel production routes, one can endogenize a portion of the hydrogen demand and evaluate its impact on the hydrogen production mix. This is an important topic for future work since it could shed light on potential future demand pathways for hydrogen that would complement the stronger existing research focus on hydrogen supply.

## Supporting Information Available

Additional national-level and regional results for the core scenarios; results from additional scenarios evaluated as part of sensitivity analysis; summary of modeling assumptions and implementation details (PDF)
