# Supplementary material for: Role of Technology Flexibility and Grid Coupling on Hydrogen Deployment in Net-Zero Energy Systems
Source: Environ Sci Technol. 2025 Mar 4;59(10):4974–88. doi: 10.1021/acs.est.4c12166 (PMC11924227; doi:10.1021/acs.est.4c12166)
Supplement: Supplementary file 1 — es4c12166_si_001.pdf [file es4c12166_si_001.pdf]

Supporting Information for

Role of technology flexibility and grid coupling on hydrogen deployment in net-zero energy systems

Jun Wen Law[1], Bryan K. Mignone[2], Dharik S. Mallapragada[3*]

1. MIT Energy Initiative, Massachusetts Institute of Technology, Cambridge, MA 02139

2. ExxonMobil Technology and Engineering Company, Annandale, NJ 08801

3. Chemical and Biomolecular Engineering Department, Tandon School of Engineering, New York University, Brooklyn, NY 11201

*Correspondence: Dharik S. Mallapragada

Email: dharik.mallapragada@nyu.edu

Summary: This supporting information contains 40 pages, 17 figures, 27 tables, and 5 equations.

Supporting Information

S1. Additional national-level results

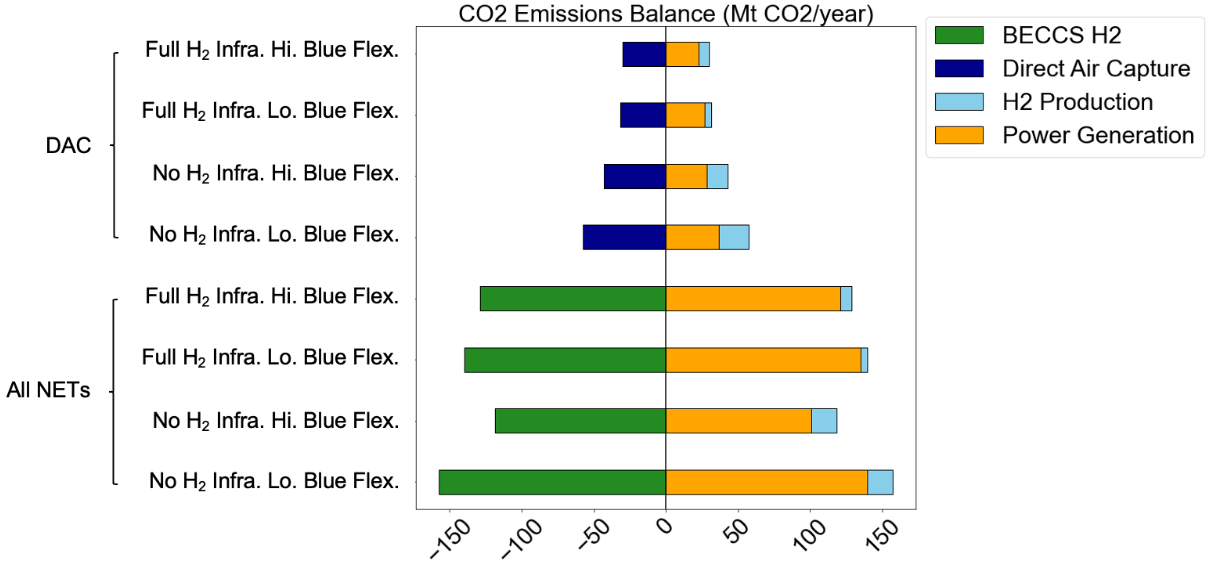

Figure S1. Emission balance of core scenarios. BECCS = bioenergy with CO_2 capture and sequestration.

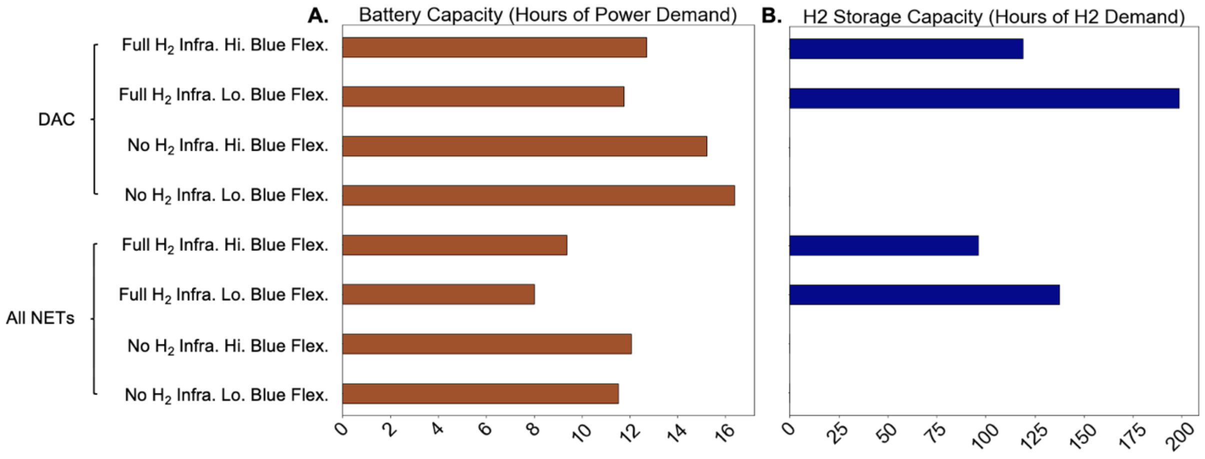

Figure S2. Installed Battery and H_2 storage capacity for the core scenarios. Storage capacity reported in units of hours of average energy demand, which refers to the ratio of installed energy storage capacity and average hourly energy (power or hydrogen) demand. Total annual power demand = 5497 TWh, which corresponds to average hourly demand of 627.5 GW. Total annual hydrogen demand = 1936 TWh, which corresponds to average hourly demand of 221 GW.

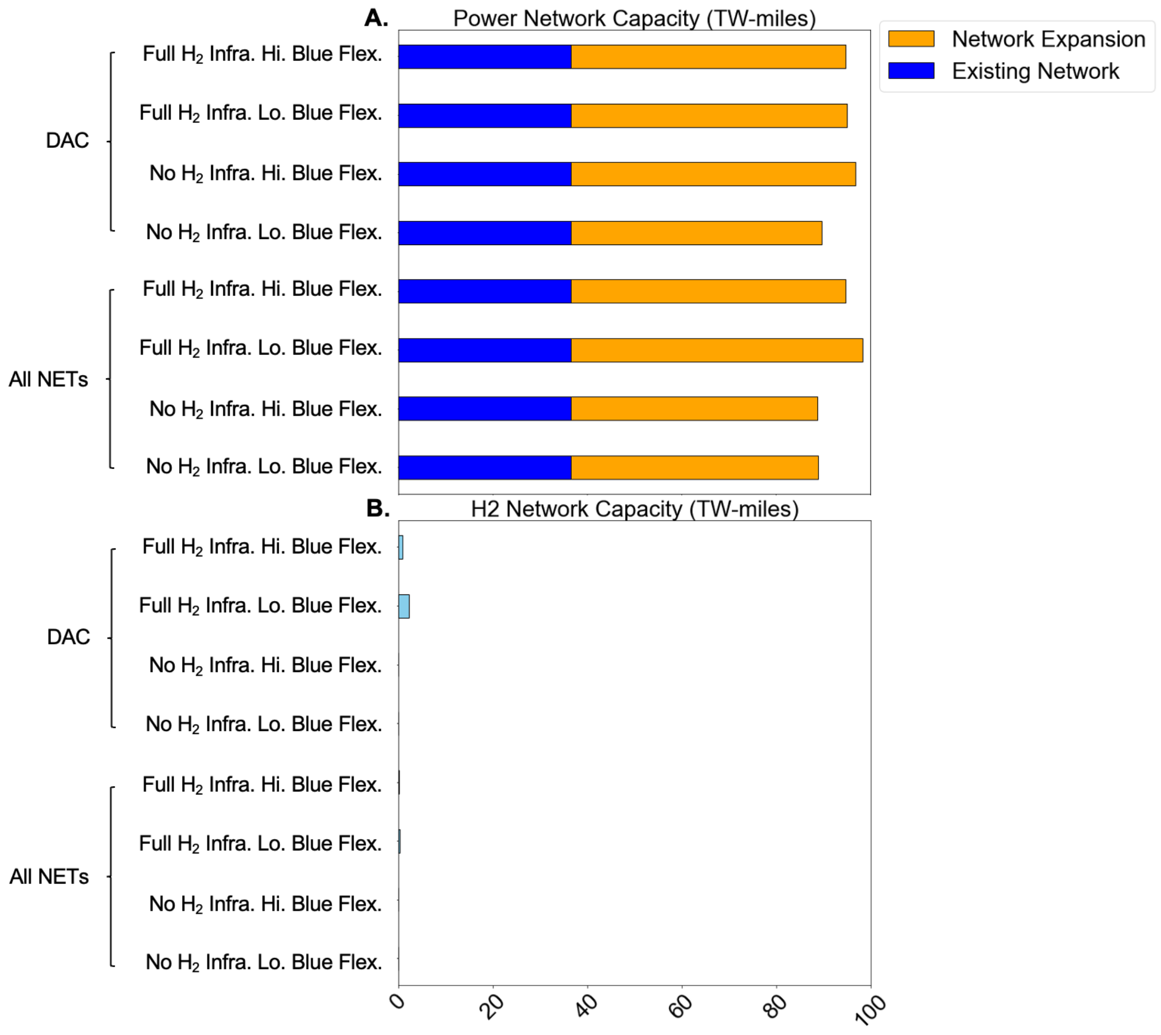

Figure S3. Power transmission and hydrogen pipeline network expansion in the core scenarios.

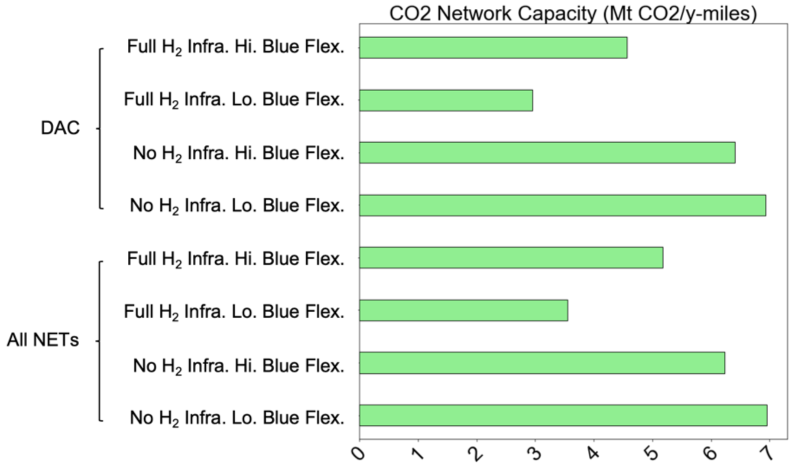

Figure S4. CO_2 pipeline network expansion in the core scenarios.

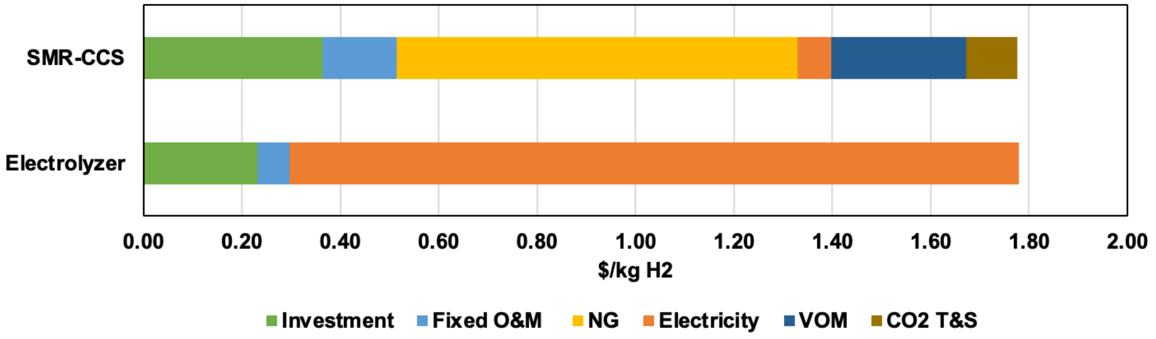

Figure S5. Comparison of cost breakdown to equalize LCOH of electrolytic and blue H_2 (SMR-CCS) with the following assumptions: Electrolyzer and SMR-CCS costs from Table S11, fuel cost of $4.39/MMBtu (corresponding to NCEN region in Table S21), assumed CO_2 transportation and storage (T&S) cost of $11/tonne CO_2, electrolyzer capacity factor of 70%, blue H_2 capacity factor of 90%, no additional costs associated with H_2 storage or residual CO_2 emissions. It is calculated that an electricity price of $33/MWh is required to equalize the LCOH of electrolytic and blue H_2. Fixed O&M = fixed operation and maintenance costs, VOM = variable operational and maintenance costs.

Table S1. CO_2 marginal abatement costs, national average electricity and H2 prices, and system average energy cost across the core scenarios. National average prices calculated using shadow price of demand-supply constraints according to according to equations in Section S7. System average energy cost calculated by dividing the total system cost by the total exogeneous energy demand.

	CO2 Marginal Abatement Cost ($/tonne CO2)		National Average Electricity Price ($/MWh)		National Average H2 Price ($/kg H2)		System Average Energy Cost ($/WMh)	
Scenarios	DAC	All NETs	DAC	All NETs	DAC	All NETs	DAC	All NETs
Full H_2 Infra. Hi. Blue Flex.	271.51	83.46	45.47	44.17	1.68	1.68	44.31	42.64
Full H_2 Infra. Lo. Blue Flex.	271.62	79.82	45.54	44.15	1.76	1.74	44.76	42.78
No H_2 Infra. Hi. Blue Flex.	271.60	80.36	47.06	45.77	1.74	1.72	45.00	43.18
No H_2 Infra. Lo. Blue Flex.	273.04	73.67	51.42	48.18	1.85	1.78	46.22	43.72

Table S2. Capacity factors for electrolyzer and blue H_2 in the core scenarios.

	Electrolyzer (%)		Blue H2 (%)	
Scenarios	DAC	All NETs	DAC	All NETs
Full H_2 Infra. Hi. Blue Flex.	74%	72%	64%	72%
Full H_2 Infra. Lo. Blue Flex.	69%	74%	87%	88%
No H_2 Infra. Hi. Blue Flex.	71%	58%	63%	69%
No H_2 Infra. Lo. Blue Flex.	75%	56%	86%	87%

S2. Additional regional results

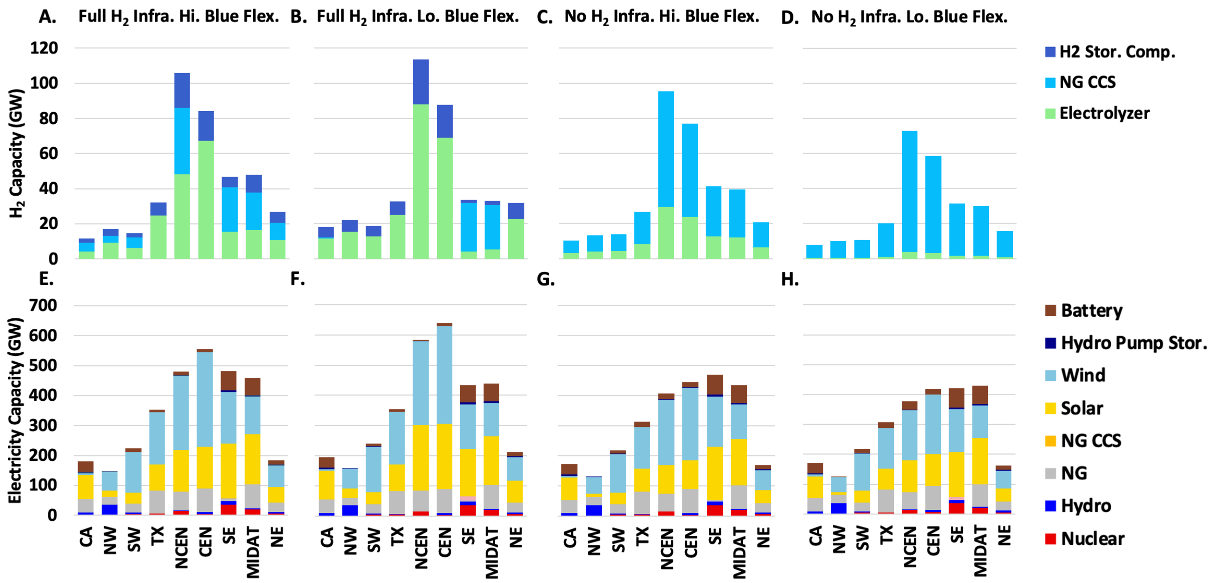

Figure S6. Regional hydrogen production and electricity generation capacity mix by technology for DAC core scenarios with different assumptions about H_2 infrastructure flexibility. The regional acronyms and their geographic location are highlighted in Figure 1C. NG = Natural Gas, CCS = CO_2 capture and sequestration, Stor. = Storage, Comp. = Compressor. Similar results for the All NETs scenarios are shown in Figure S8.

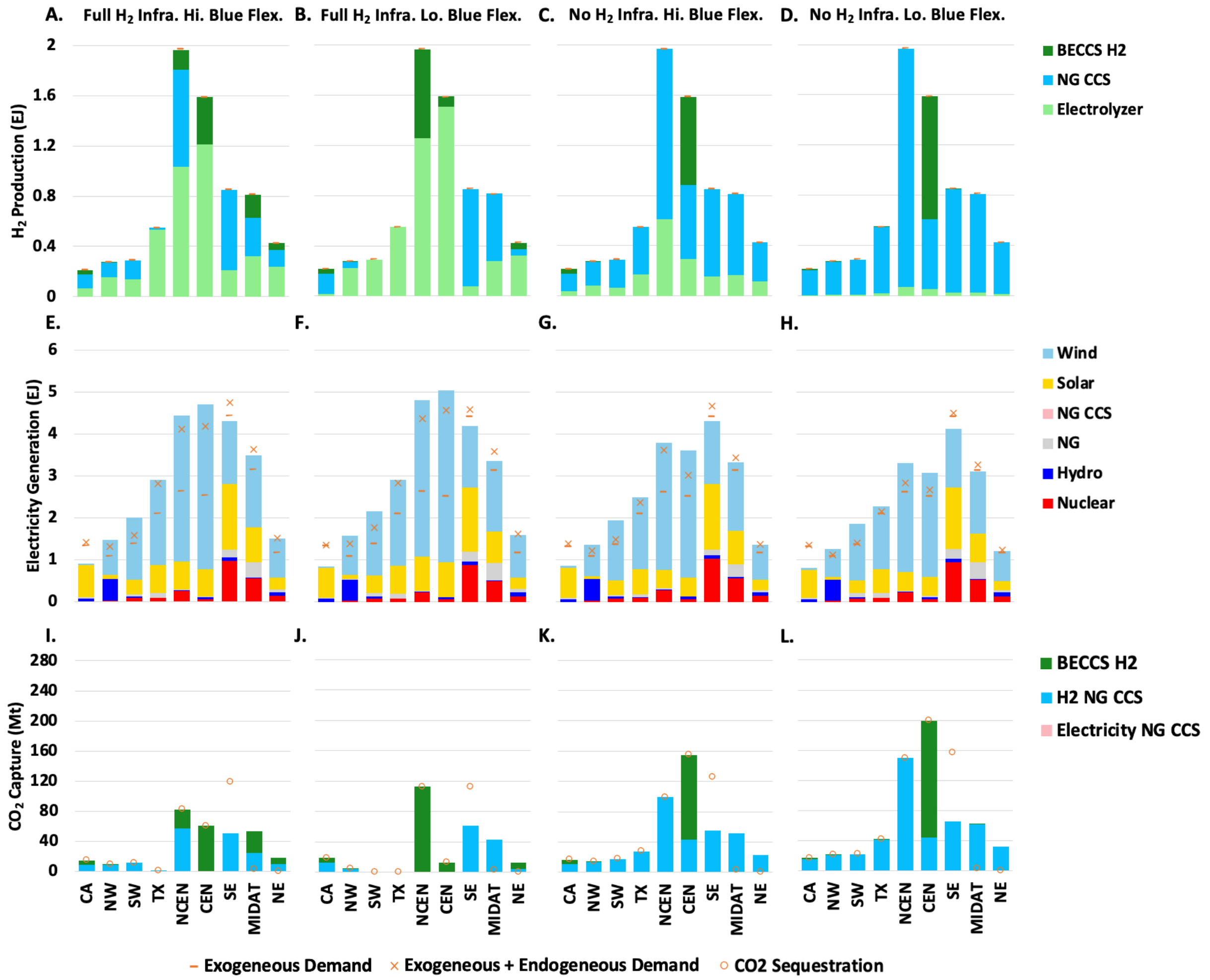

Figure S7. Regional electricity generation, hydrogen production, and CO_2 captured by technology with different assumptions about hydrogen infrastructure flexibility and blue H_2 operational flexibility. Results are shown for the All NETs scenarios. The orange line on each bar indicates exogeneous demand (for both electricity and hydrogen) in each region, and the orange "x" indicates the total (exogeneous plus endogenous) electricity demand in each region. If the bars are lower than the orange markers in a given region, then that region is a net electricity or hydrogen importer, and vice versa. There are losses associated with electricity transmission and storage, which implies that total generation will exceed demand. Orange circles in the bottom panel correspond to CO_2 sequestered in each region. When CO_2 sequestered exceeds CO_2 captured in a given region, then that region is a net importer of captured CO_2, and vice versa. The regional acronyms are defined and their geographic locations are shown in Figure 1C. Similar results for the DAC scenarios are shown in Figure 3. BECCS = bioenergy with CO_2 capture and sequestration.

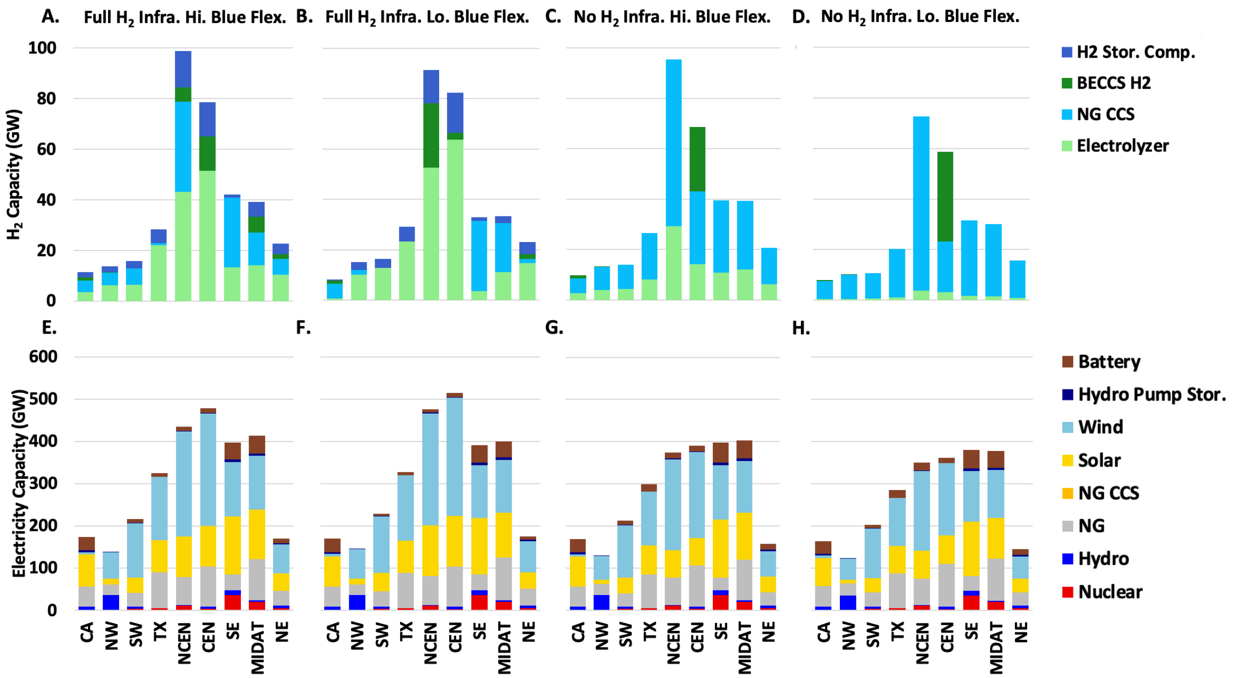

Figure S8. Regional hydrogen production and electricity generation capacity mix by technology for All NETs core scenarios with different assumptions about H_2 infrastructure flexibility. The regional acronyms and their geographic location are highlighted in Figure 1C. BECCS = bioenergy with CO_2 capture and sequestration, NG = Natural Gas, CCS = CO_2 capture and sequestration, Stor. = Storage, Comp. = Compressor.

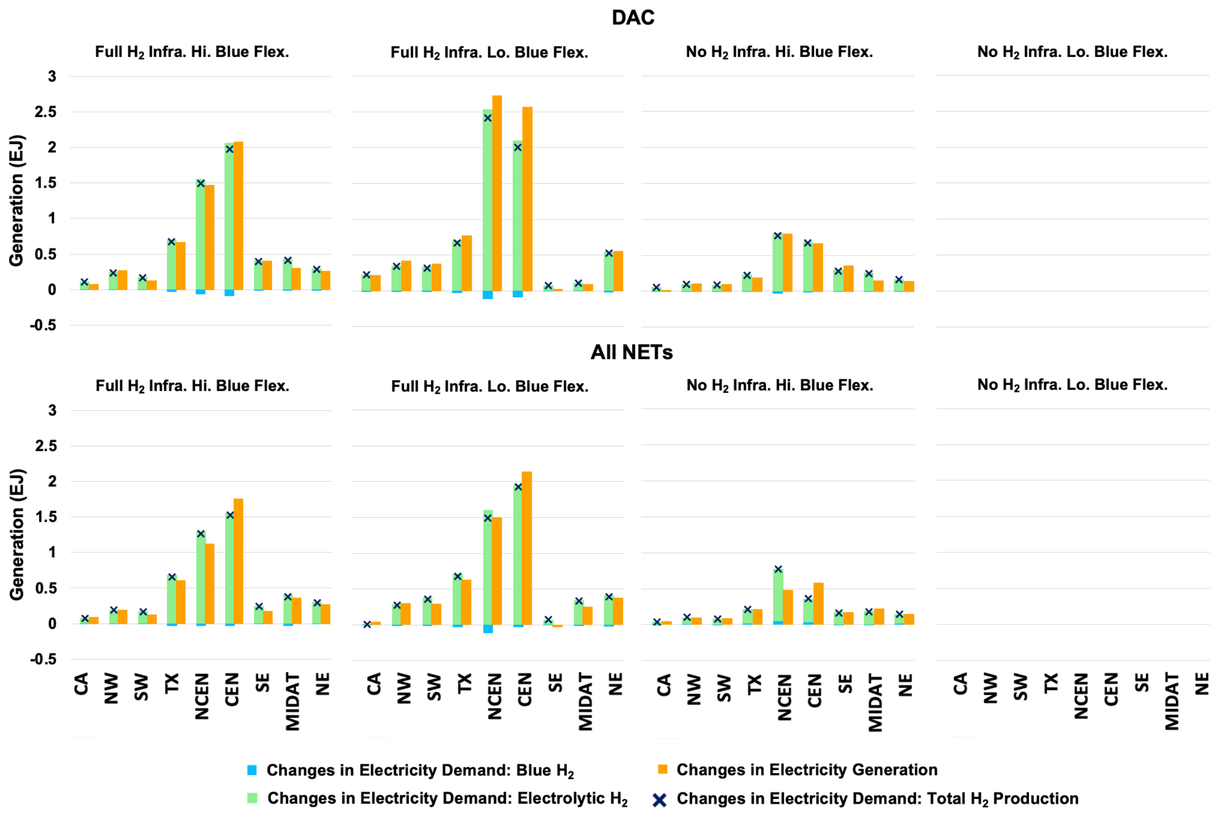

Figure S9. Regional changes in electricity demand compared with changes in electricity supply, where all changes are compared to the case with the least electrolytic H_2 (No. H_2 Infra. Lo. Blue Flex.), for different assumptions about H_2 infrastructure flexibility and NETs. The electrolyzer electricity demand is calculated by dividing the electrolytic H_2 produced in each region by the electrolyzer conversion efficiency (74%). The regional acronyms and their geographic location are highlighted in Figure 1C.

Tabular results for electricity prices calculated using equations in Section S7.

Table S3. Summary of regional avg. electricity price (Elec. price), electricity supply cost for electrolyzer (Elec. Supply cost) and regional share of national H_2 production (%) for the scenarios of full H_2 infrastructure and high blue H_2 flexibility, and full H_2 infrastructure with low blue H_2 flexibility with DAC as the negative emissions technology. National electrolyzer share (%) represent the percentage of electrolysis H_2 in satisfying the national H_2 demand. Similar results for other core scenarios in Table S4-S6. The electricity price reported is the sum of the shadow value of the hourly supply-demand balance constraint and the planning reserve margin constraint, corresponding to the energy and capacity components of the electricity price, respectively.

Region	Full H_2 Infra. Hi. Blue Flex. (DAC)					Full H_2 Infra. Lo. Blue Flex. (DAC)				
	Elec. Price ($/MWh)	Elec. supply cost ($/MWh)	Ely. Share of total H_2 production (%)	Ely. Capacity (GW)	Ely. Capacity Factor	Elec. Price ($/MWh)	Elec. supply cost ($/MWh)	Ely. Share of total H_2 production (%)	Ely. Capacity (GW)	Ely. Capacity Factor
CA	53.26	24.71	1.27%	4.1	0.68	53.18	32.73	2.60%	11.4	0.51
NW	42.65	25.80	2.76%	9.1	0.67	42.15	30.58	3.94%	15.3	0.57
SW	42.49	20.40	2.05%	6.3	0.71	42.52	26.70	3.73%	12.8	0.64
TX	36.16	21.62	7.87%	24.8	0.70	35.89	21.79	7.86%	25.0	0.70
NCEN	43.11	23.96	17.91%	48.2	0.82	41.70	31.15	28.20%	88.1	0.71
CEN	38.87	26.18	23.07%	67.2	0.76	38.64	26.07	23.39%	69.1	0.75
MIDAT	47.17	18.34	4.89%	15.4	0.70	46.31	22.88	1.36%	3.9	0.77
SE	49.99	22.56	5.08%	16.5	0.68	48.65	26.31	1.71%	5.3	0.72
NE	51.73	25.08	3.41%	10.8	0.69	53.25	35.34	6.12%	22.6	0.60
National	45.47	23.97	68.31%	202.5	0.74	45.54	28.60	78.91%	253.5	0.69

Table S4. Summary of regional avg. electricity price (Elec. price), electricity supply cost for electrolyzer (Elec. Supply cost) and regional share of national H_2 production (%) for the scenarios of no H_2 storage & no H_2 pipelines (low blue H_2 flexibility), and no H_2 storage & no H_2 pipelines (inflexibility blue H_2), with DAC as the negative emissions technology. National electrolyzer share (%) represent the percentage of electrolysis H_2 in satisfying the national H_2 demand.

Region	No H_2 Infra. Hi. Blue Flex. (DAC)					No H_2 Infra. Lo. Blue Flex. (DAC)				
	Elec. Price ($/MWh)	Elec. supply cost ($/MWh)	Ely. Share of total H_2 production (%)	Ely. Capacity (GW)	Ely. Capacity Factor	Elec. Price ($/MWh)	Elec. supply cost ($/MWh)	Ely. Share of total H_2 production (%)	Ely. Capacity (GW)	Ely. Capacity Factor
CA	53.09	17.91	0.76%	3.2	0.53	53.40	14.52	0.13%	0.4	0.68
NW	42.13	16.00	1.24%	4.1	0.67	43.17	10.25	0.17%	0.5	0.71
SW	42.22	12.33	1.17%	4.3	0.60	42.52	9.06	0.18%	0.6	0.71
TX	36.54	8.83	2.82%	8.2	0.76	35.78	6.48	0.39%	1.1	0.81
NCEN	42.16	15.55	9.93%	29.4	0.75	42.44	10.55	1.32%	3.8	0.76
CEN	39.20	13.43	8.51%	23.7	0.79	39.01	8.61	1.10%	3.1	0.79
MIDAT	46.38	15.41	3.68%	12.7	0.64	46.07	12.48	0.53%	1.7	0.71
SE	49.01	16.33	3.25%	12.1	0.59	48.61	13.92	0.50%	1.6	0.70
NE	49.56	17.39	2.05%	6.4	0.71	49.70	14.23	0.28%	0.8	0.73
National	47.06	14.57	33.42%	104.1	0.71	51.42	10.59	4.60%	13.6	0.75

Table S5. Summary of regional avg. electricity price (Elec. price), electricity supply cost for electrolyzer (Elec. Supply cost) and regional share of national H_2 production (%) for the scenarios of full H_2 infrastructure and high blue H_2 flexibility, and full H_2 infrastructure with low blue H_2 flexibility with All NETs as the negative emissions technology. National electrolyzer share (%) represent the percentage of electrolysis H_2 in satisfying the national H_2 demand.

Region	Full H_2 Infra. Hi. Blue Flex. (All NETs)					Full H_2 Infra. Lo. Blue Flex. (All NETs)				
	Elec. Price ($/MWh)	Elec. supply cost ($/MWh)	Ely. Share of total H_2 production (%)	Ely. Capacity (GW)	Ely. Capacity Factor	Elec. Price ($/MWh)	Elec. supply cost ($/MWh)	Ely. Share of total H_2 production (%)	Ely. Capacity (GW)	Ely. Capacity Factor
CA	51.60	23.77	1.03%	3.43	0.59	51.13	23.52	0.22%	0.70	0.61
NW	41.45	26.04	2.52%	6.3	0.78	41.14	31.49	3.64%	10.1	0.70
SW	40.83	22.49	2.28%	6.5	0.69	40.42	29.83	4.75%	12.8	0.72
TX	35.93	25.09	8.62%	22.2	0.76	36.00	25.32	9.00%	23.3	0.75
NCEN	39.86	24.87	16.77%	43.2	0.76	39.62	29.50	20.69%	52.7	0.76
CEN	37.69	26.02	19.67%	51.5	0.75	37.77	28.08	24.74%	63.7	0.75
MIDAT	44.09	18.81	3.41%	13.4	0.50	43.99	25.45	1.30%	3.6	0.70
SE	45.83	28.08	5.23%	14.2	0.72	45.69	31.34	4.59%	11.2	0.79
NE	49.93	30.99	3.85%	10.5	0.72	49.82	35.74	5.25%	14.6	0.69
National	44.17	25.51	63.39%	171.2	0.72	44.15	29.11	74.18%	192.6	0.74

Table S6. Summary of regional avg. electricity price (Elec. price), electricity supply cost for electrolyzer (Elec. Supply cost) and regional share of national H_2 production (%) for the scenarios of no H_2 storage & no H_2 pipelines (low blue H_2 flexibility), and no H_2 storage & no H_2 pipelines (inflexibility blue H_2), with All NETs as the negative emissions technology. National electrolyzer share (%) represent the percentage of electrolysis H_2 in satisfying the national H_2 demand.

Region	No H_2 Infra. Hi. Blue Flex. (All NETs)					No H_2 Infra. Lo. Blue Flex. (All NETs)				
	Elec. Price ($/MWh)	Elec. supply cost ($/MWh)	Ely. Share of total H_2 production (%)	Ely. Capacity (GW)	Ely. Capacity Factor	Elec. Price ($/MWh)	Elec. supply cost ($/MWh)	Ely. Share of total H_2 production (%)	Ely. Capacity (GW)	Ely. Capacity Factor
CA	50.98	17.79	0.62%	2.64	0.46	51.05	14.39	0.10%	0.39	0.46
NW	41.40	17.00	1.29%	4.1	0.63	41.21	10.84	0.15%	0.5	0.52
SW	40.16	12.55	1.09%	4.3	0.50	40.01	10.06	0.15%	0.6	0.52
TX	34.93	10.98	2.73%	8.2	0.66	34.78	8.23	0.36%	1.1	0.63
NCEN	39.93	17.68	9.81%	29.4	0.66	38.90	11.53	1.22%	3.8	0.60
CEN	37.11	13.68	4.75%	14.3	0.66	37.08	9.15	0.95%	3.1	0.58
MIDAT	44.12	17.88	2.44%	10.8	0.45	43.13	14.11	0.42%	1.7	0.48
SE	45.62	17.63	2.64%	12.1	0.43	44.64	14.39	0.40%	1.6	0.49
NE	49.28	20.28	1.83%	6.4	0.57	48.95	15.12	0.23%	0.8	0.52
National	45.77	16.26	27.20%	92.2	0.58	48.18	11.43	3.97%	13.5	0.56

S3. Additional results related to system operation

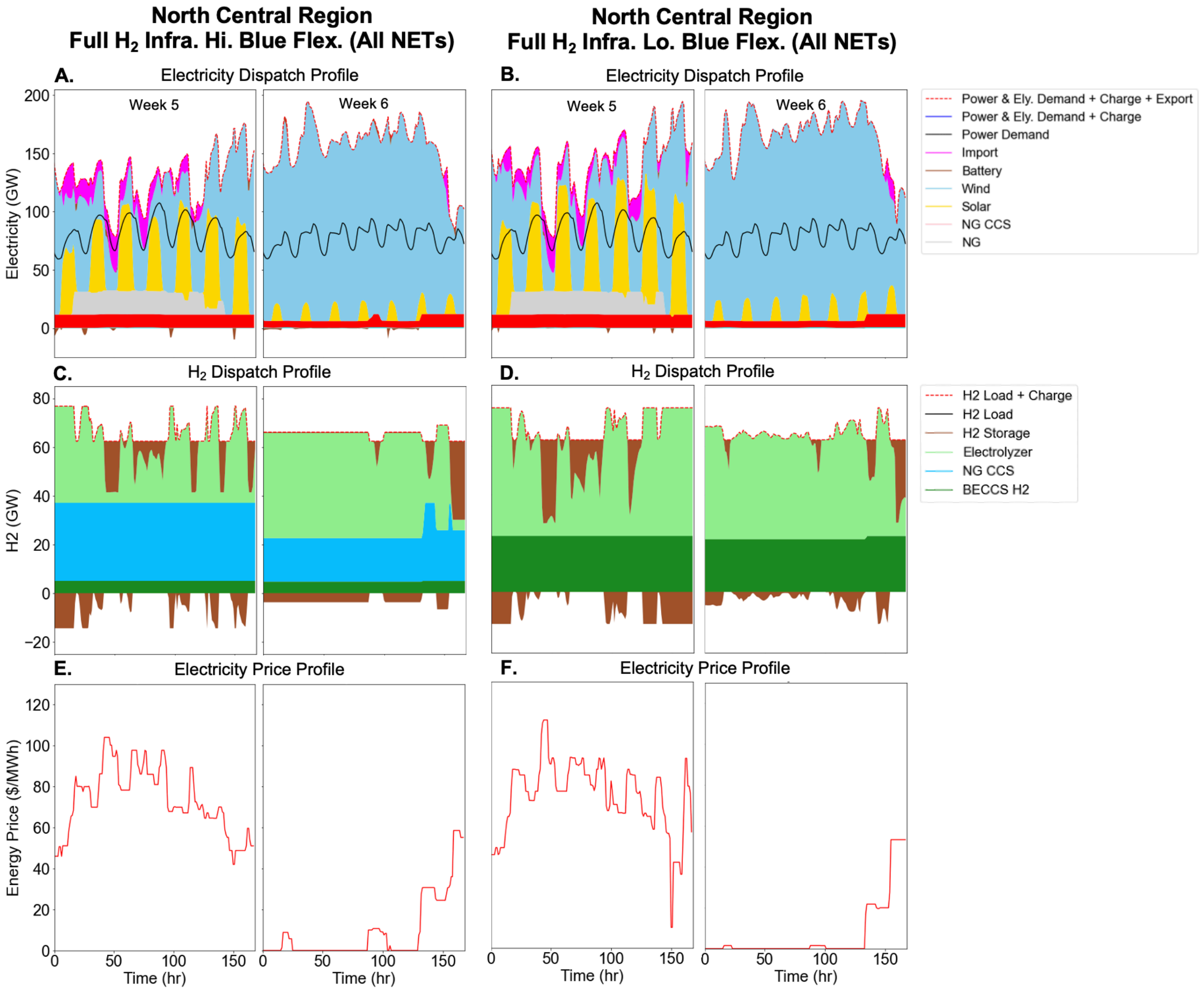

Figure S10. System operation for electricity (Panel A and B) and hydrogen (Panel C and D) production sectors, as well as electricity prices (Panel E and F) for two representative weeks in the north central (NCEN) region. All NETs scenarios with full hydrogen infrastructure are shown, with high blue H_2 flexibility on plots A,C,E, and low blue H_2 flexibility on plots B,D,F. The left half of each panel shows a representative week in summer with limited wind but abundant solar resources, whereas the right half of each panel shows a representative week in winter when the wind resource is abundant but solar is limited. Panel E and F plot the energy component of electricity prices (left y-axis) using the dual variable on the hourly supply-demand balance constraint. Similar results for the DAC scenarios are shown in Figure 4. Ely = Electrolyzer.

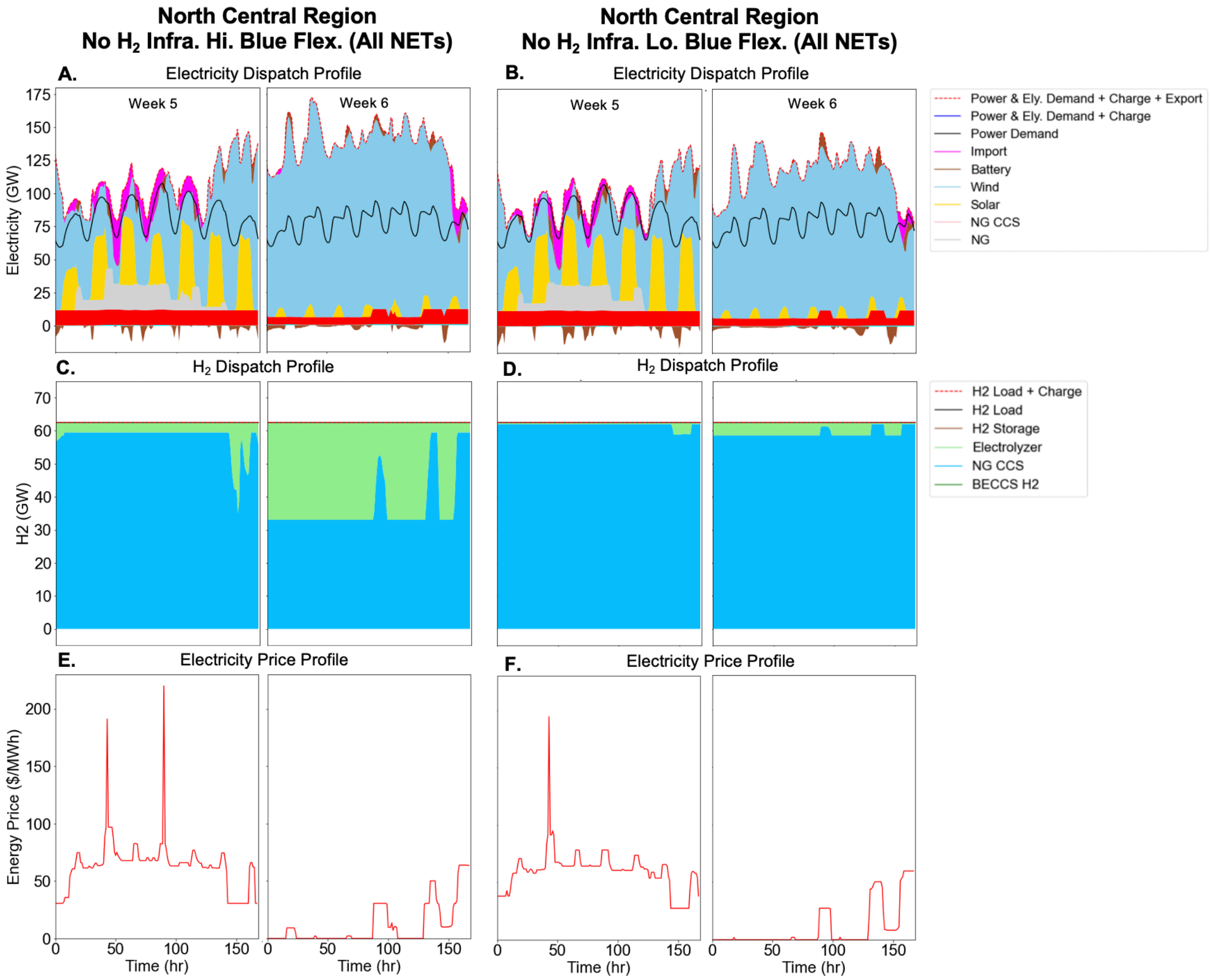

Figure S11. Dispatch profiles for electricity (Panel A and B) and hydrogen (Panel C and D) production sectors, as well as electricity prices (Panel E and F) for two representative weeks in the north central (NCEN) region. All NETs scenarios with no hydrogen storage and pipeline infrastructure are shown, with high blue H_2 flexibility on plots A,C,E, and low blue H_2 flexibility on plots B,D,F. The left half of each panel shows a representative week in summer with limited wind but abundant solar resources, whereas the right half of each panel shows a representative week in winter when the wind resource is abundant but solar is limited. Panel E and F plot the energy component of electricity prices (left y-axis) using the dual variable on the hourly supply-demand balance constraint. Similar results for the DAC scenarios are shown in Figure 5. Ely = Electrolyzer.

S4. Additional results related to grid-connected vs islanded H_2 production

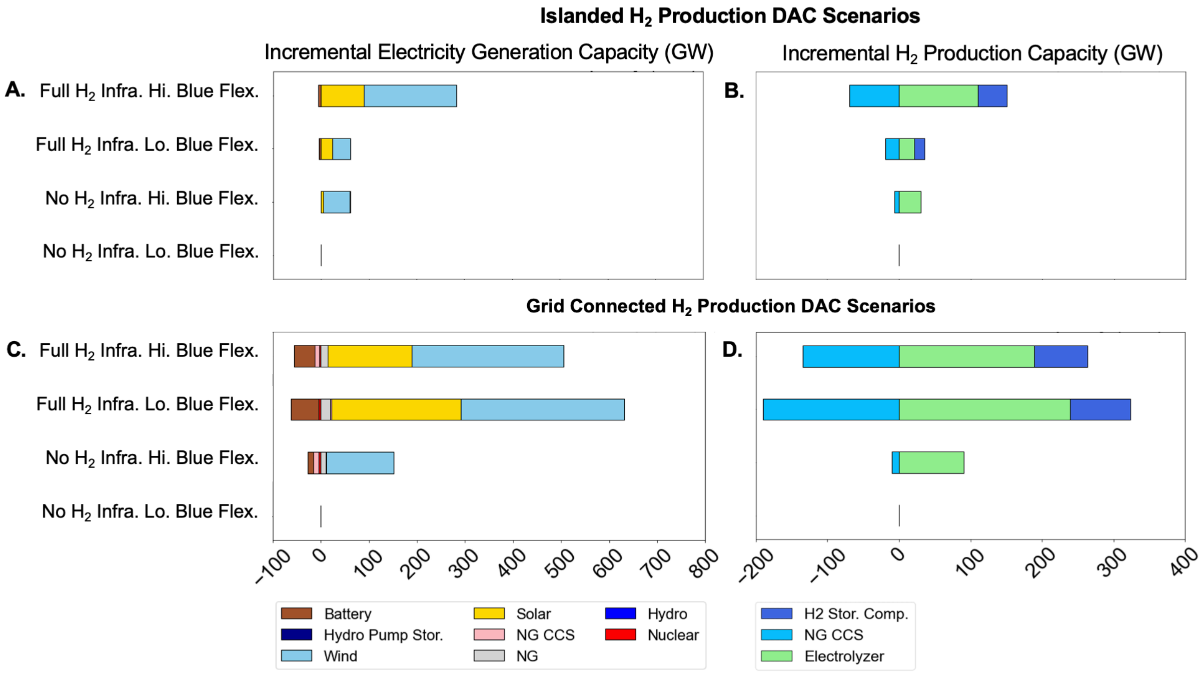

Figure S12. Incremental electricity generation capacity and incremental hydrogen production capacity compared to the least flexible hydrogen infrastructure scenario (no H_2 infrastructure + Lo Blue Flex.) for DAC scenarios under islanded and grid-connected systems. The islanded system is differentiated from the grid-connected system in the following ways: (a) no exogeneous power demand and no existing power generation, storage or transmission capacity, (b) only VRE and battery storage capacity can be added in the power sector, and (c) no capacity reserve margin constraint. Grid-connected hydrogen production scenarios are identical to those in Figure 2. NG = Natural Gas, CCS = CO_2 capture and sequestration, Stor. = Storage, Comp. = Compressor.

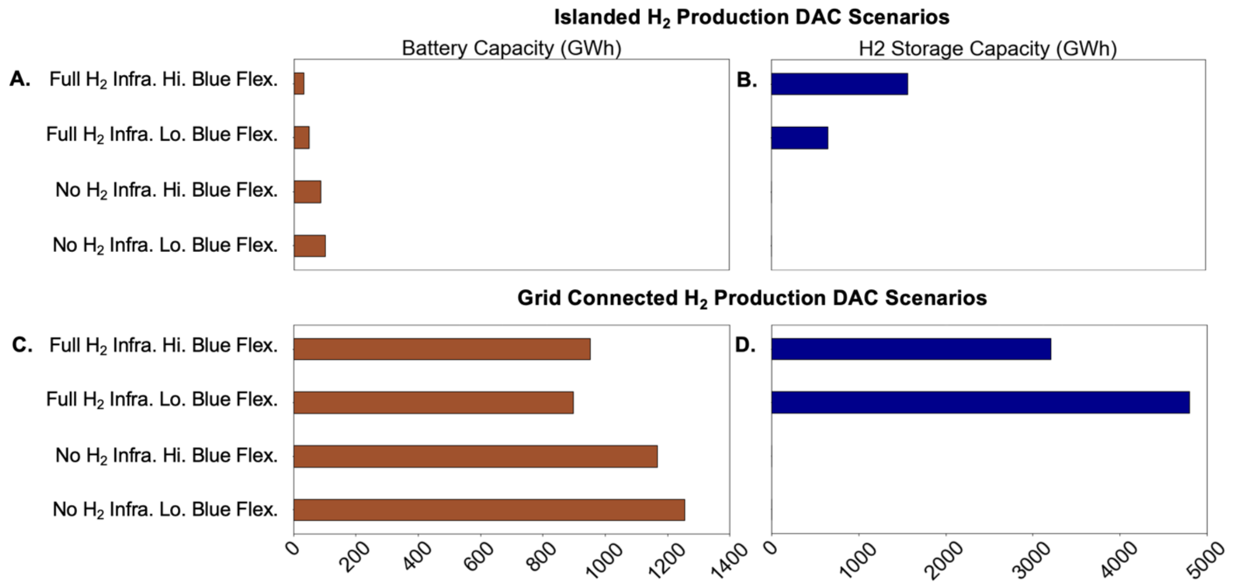

Figure S13. Absolute power and hydrogen energy storage capacity of DAC scenarios under islanded (panels A,B) and grid connected H_2 production (panels C,D).

Table S7. Electrolyzer and blue H_2 capacity factors of grid-connected versus islanded systems for DAC scenarios. VRE curtailment is defined as fraction of available VRE generation that is not dispatched.

	Electrolyzer (%)		Blue H_2 (%)		VRE Curtailment (%)	
Scenarios	Grid-connected	Islanded	Grid-connected	Islanded	Grid-connected	Islanded
Full H_2 Infra. Hi. Blue Flex.	69%	77%	64%	72%	6.2%	3.8%
Full H_2 Infra. Lo. Blue Flex.	74%	78%	87%	87%	7.0%	5.7%
No H_2 Infra. Hi. Blue Flex.	71%	73%	63%	80%	4.8%	2.0%
No H_2 Infra. Lo. Blue Flex.	75%	77%	86%	87%	8.1%	4.6%

Table S8. Solar and wind share of total electricity generation grid-connected vs islanded systems for DAC scenarios.

	Solar (%)		Wind (%)	
Scenarios	Grid-connected	Islanded	Grid-connected	Islanded
Full H_2 Infra. Hi. Blue Flex.	26%	21%	63%	79%
Full H_2 Infra. Lo. Blue Flex.	27%	25%	62%	74%
No H_2 Infra. Hi. Blue Flex.	24%	15%	62%	84%
No H_2 Infra. Lo. Blue Flex.	26%	25%	59%	73%

S5. Results from sensitivity cases

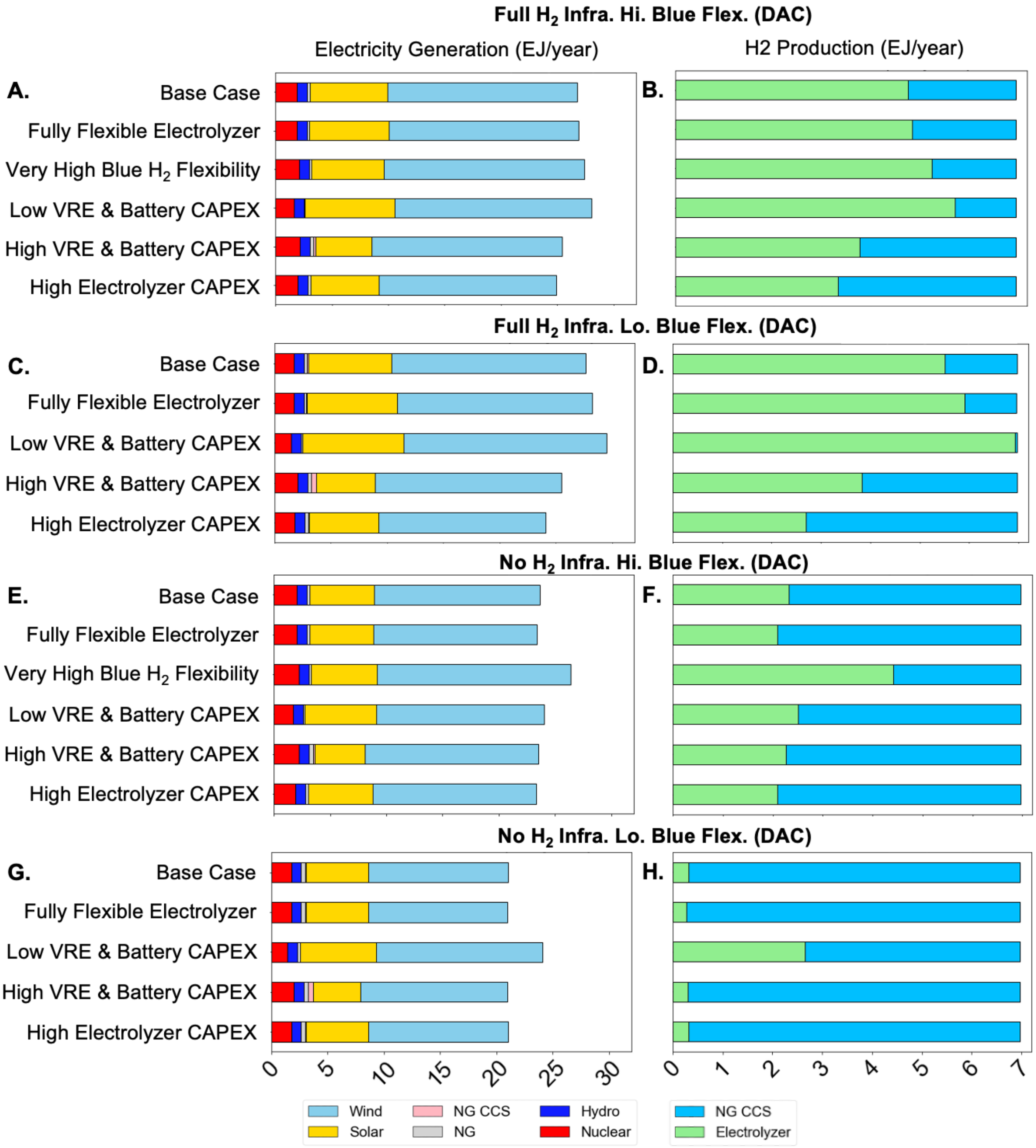

Figure S14. Sensitivity of electricity generation and hydrogen production mix to several assumptions based on the core DAC cases. Sensitivity cases include: Low VRE and battery CAPEX obtained from 2045 costs (advanced) reported by the NREL Annual Technology Baseline 2021 [1], High VRE and battery CAPEX obtained from 2045 costs (conservative) reported by the NREL Annual Technology Baseline 2021 [1] high electrolyzer CAPEX of $900/kWe per IEA assumptions ("Today" scenario CAPEX) [2], very high blue H_2 flexibility (between 10-90% of nameplate capacity), fully flexible electrolyzer (between 0-100% of nameplate capacity). "Base case" refers to the results reported for the core scenarios in Figures 2-5 in the main text. NG = Natural Gas, CCS = CO_2 capture and sequestration.

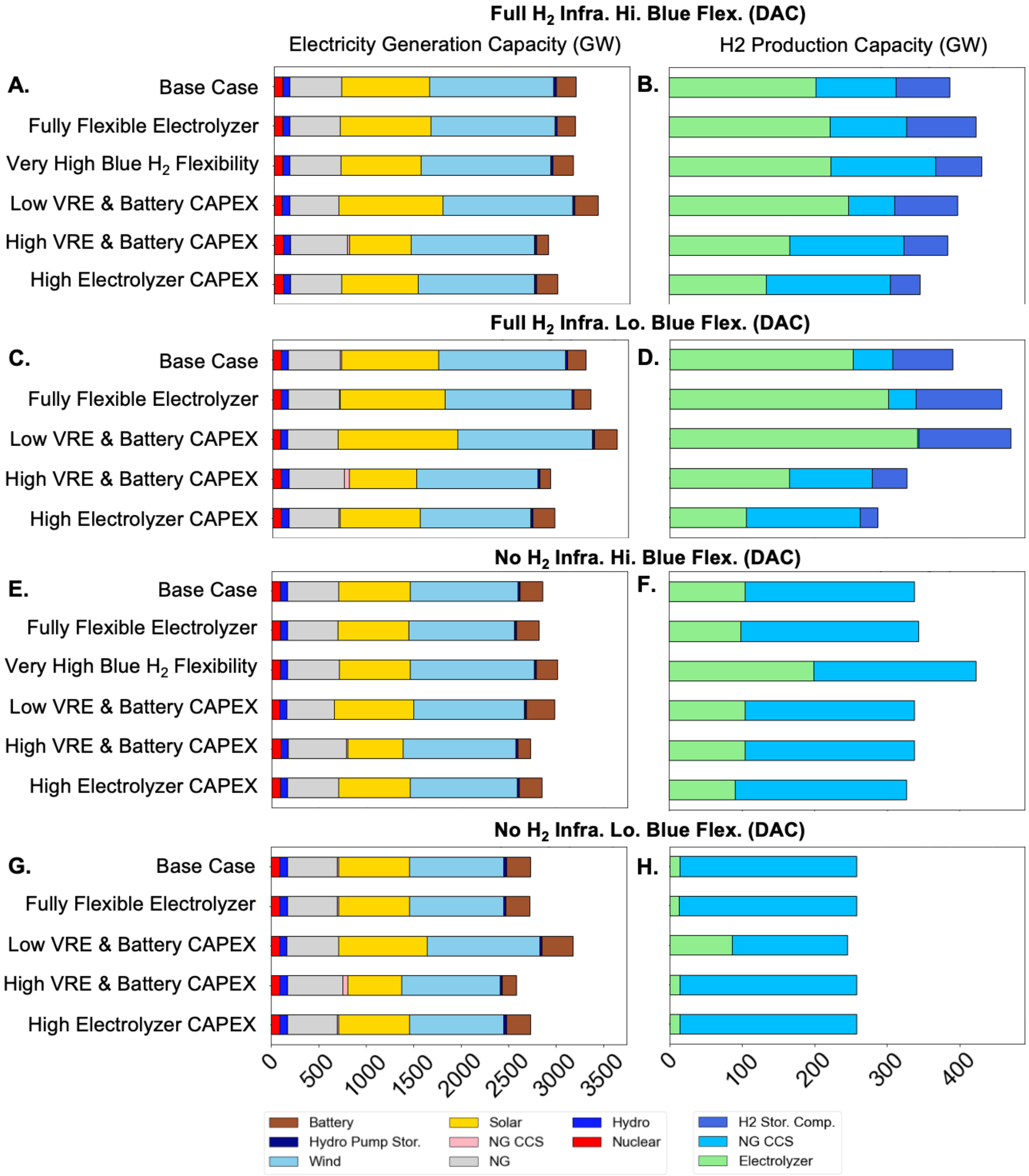

Figure S15. Sensitivity of electricity generation and hydrogen production capacity mix to several assumptions based on the core DAC cases. Sensitivity cases include: Low VRE and battery CAPEX obtained from 2045 costs (advanced) reported by the NREL Annual Technology Baseline 2021 [1], High VRE and battery CAPEX obtained from 2045 costs (conservative) reported by the NREL Annual Technology Baseline 2021 [1] high electrolyzer CAPEX of $900/kWe per IEA assumptions ("Today" scenario CAPEX) [2], very high blue H_2 flexibility (between 10-90% of nameplate capacity), fully flexible electrolyzer (between 0-100% of nameplate capacity). "Base case" refers to the results reported for the core scenarios in Figures 2-5 in the main text. NG = Natural Gas, CCS = CO_2 capture and sequestration.

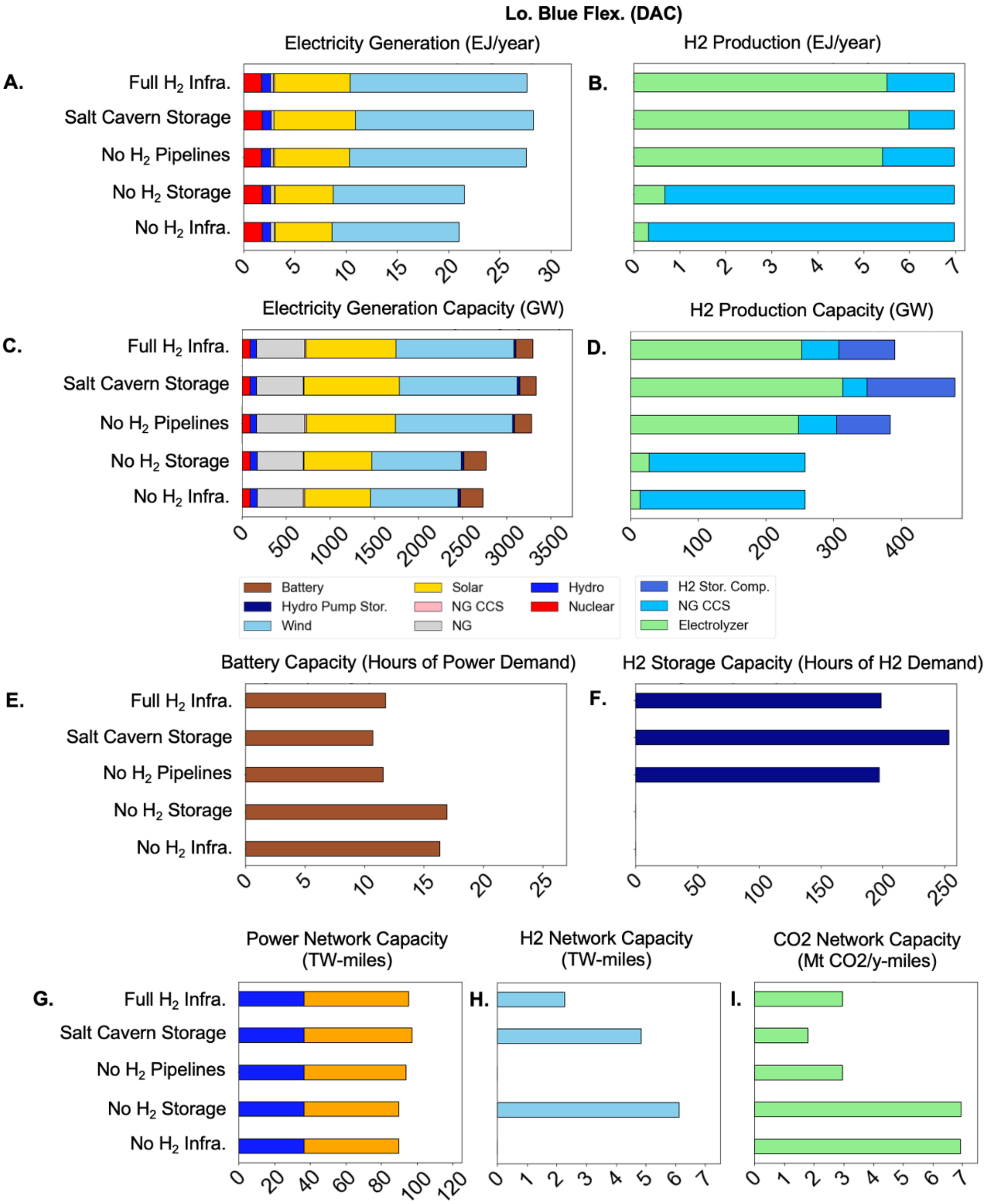

Figure S16. Sensitivity of electricity generation and hydrogen production mix, capacity, energy storage, and transmission to alternative infrastructure assumptions based on the core DAC scenarios. "Full H_2 Infra" and "No H_2 Infra" cases correspond to the core cases with low blue H_2 flexibility. "Salt Cavern Storage" refers to the addition of low-cost geological H2 storage (Table S11) in TX, SW, and NCEN regions. The two other cases separately vary the availability of H_2 storage and H_2 pipelines, also with low blue H_2 flexibility. NG = Natural Gas, CCS = CO_2 capture and sequestration.

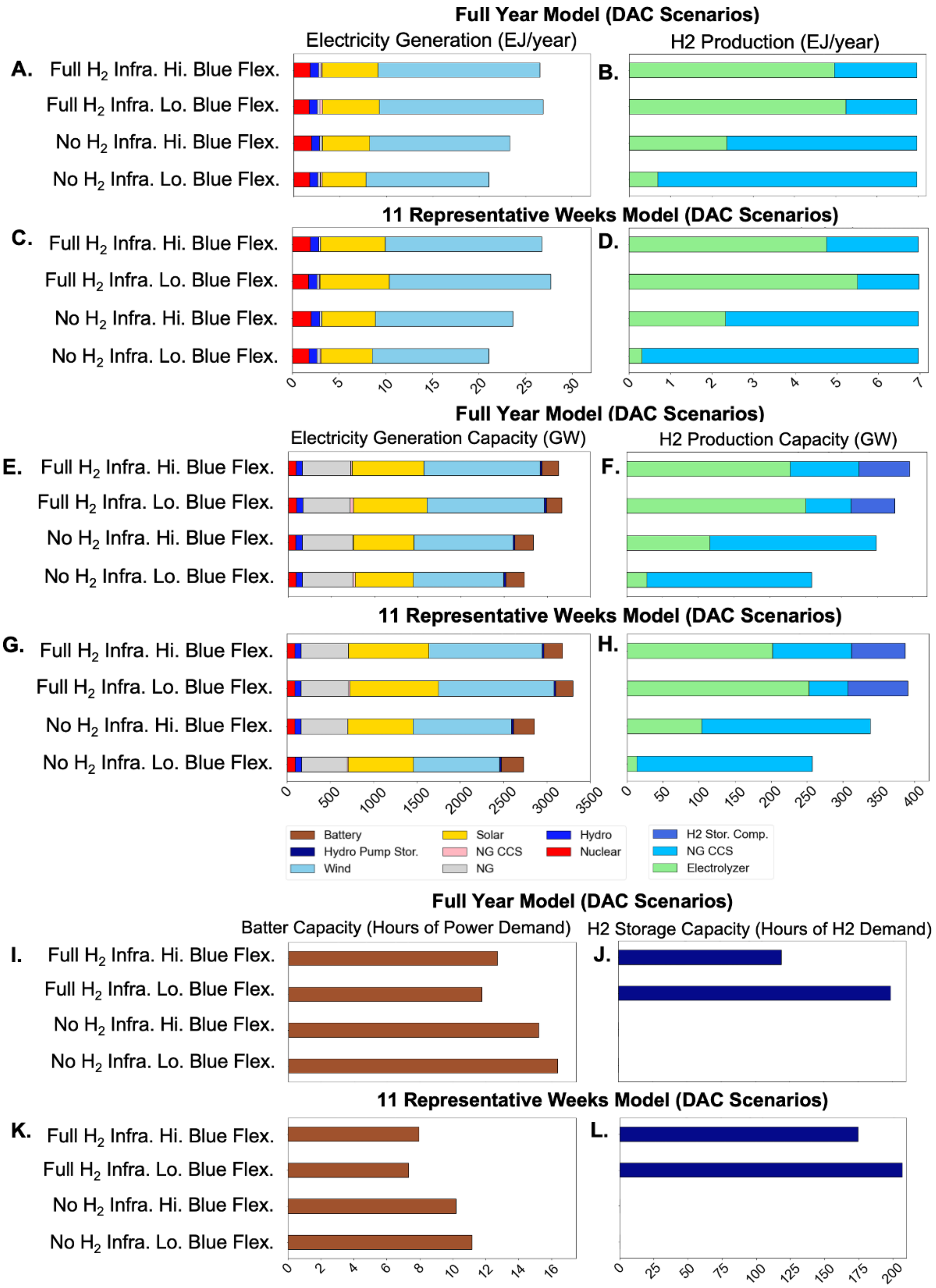

Figure S17. Comparison of electricity generation and hydrogen production mix, capacity, and energy storage results between 11 representative weeks of system operation vs. one year of operations at hourly resolution (Full Year Model).

S6. Modeling assumptions

Table S9. Existing capacity and cost parameters for network expansion for power network transmission lines obtained from the U.S. Environmental Protection Agency (EPA) version of the Integrated Planning Model (IPM) [3].

Network Lines	Transmission Path	Distance (Miles)	Existing Capacity (MW)	Line Reinforcement Cost ($/MW-mile)	Annualized Line Reinforcement Cost ($/MW-mile/y)
1	CA to NW	622	6,533	1,866	115
2	CA to SW	490	11,964	1,866	115
3	NW to SW	577	4,530	1,148	70
4	SW to CEN	732	610	1,318	81
5	TX to CEN	494	2,525	1,318	81
6	NCEN to CEN	485	9,851	1,127	69
7	NCEN to SE	890	3,745	829	51
8	NCEN to MIDAT	751	9,083	1,127	69
9	CEN to SE	780	4,872	1,190	73
10	SE to MIDAT	491	5,552	1,190	73
11	MIDAT to NE	474	1,915	1,913	117

Table S10. Greenfield power generation technology cost and performance parameters. Data corresponds to 2045 costs (moderate) reported by the NREL Annual Technology Baseline 2021 in 2019 dollars [1], with the assumption that 2050 energy system would consist of technologies built five years earlier. A discount rate of 4.5% is used to annualize investment costs according to assumed technology lifetime. CC = Combined cycle, CT = Combustion turbine, CCS = CO_2 capture and storage.

Technology	Lifetime (year)	Investment cost		Annualized CAPEX		Fixed operation and maintenance (FOM) cost		Variable operation and maintenance (VOM) cost ($/MWh)	Heat Rate (MMBtu/MWh)
		Power ($/kW)	Energy ($/kWh)	Power ($/kW/year)	Energy ($/kWh/year)	Power ($/kW/year)	Energy ($/kWh/year)		
Natural Gas (CC)	15	935	-	87	-	27	-	1.74	6.36
Natural Gas (CC-CCS)	20	2,167	-	167	-	65	-	5.73	7.16
Natural Gas (CT)	15	780	-	73	-	21	-	4.94	9.72
Nuclear	40	6,152	-	334	-	145	-	2.35	10.46
Solar	20	672	-	52	-	15	-	-	-
Land Based Wind	20	808	-	62	-	35	-	-	-
Offshore Wind	20	2,928	-	225	-	56	-	-	-
Battery	15	185	113	17	11	5	3	-	-
Solar (Low CAPEX)	20	520	-	40	-	15	-	-	-

Onshore Wind (Low CAPEX)	20	569	-	44	-	35	-	-	-
Offshore Wind (Low CAPEX)	20	2,410	-	185	-	56	-	-	-
Battery (Low CAPEX)	15	76	81	7	8	5	3	-	-
Solar (High CAPEX)	20	878	-	54	-	15	-	-	-
Onshore Wind (High CAPEX)	20	925	-	57	-	35	-	-	-
Offshore Wind (High CAPEX)	20	4054	-	249	-	56	-	-	-
Battery (High CAPEX)	15	207	193	19	18	5	3	-	-

Table S11. H_2 production technology cost and performance parameters. Cost and performance parameters for electrolyzer obtained from IEA ("Long term" scenario assumption used for default inputs, and investment cost from "Today" scenario for high electrolyzer CAPEX sensitivity case in Figure S14-S15) [2], data for natural gas reforming technologies obtained from NETL techno-economic analysis study [4], and data for tank storage and salt cavern geological storage (For exemplary case in Figure S16) are obtained from Papadias & Aluwalia [5]. Feedwater cost for electrolyzers assumed to be negligible compared to the cost of electricity. The capital cost for H_2 storage in terms of rate of production and electricity input are assoicated with charging components. SMR = Steam methane reforming, ATR = Autothermal reforming, CCS = CO_2 capture and storage. Units of \$/MWH_2 are converted based on lower heating value of H_2. A discount rate of 4.5% is used to annualize investment costs and costs are converted to 2019 dollars.

Technology	Lifetime (year)	Investment cost		Annualized CAPEX		Fixed operation and maintenance (FOM) cost		Variable operation and maintenance (VOM) cost ($/t H_2)	Electricity input (MWh/t H_2)	Fuel input (MMBtu /t H_2)
		H_2 production rate ($/MW$H_2$)	Energy ($/t H_2)	H_2 production rate ($/MW$H_2$/year)	Energy ($/t H_2/year)	H_2 production rate ($/MW$H_2$/year)	Energy ($/t H_2/year)			
Electrolyzer	20	632,432	-	48,619	-	1,245	-	-	45.00	-
Electrolyzer (High CAPEX)	20	1,264,864	-	97,238	-	2,490	-	-	45.00	-
H_2 Storage (tank)	30	62,012	560,000	3,807	34,379	-	1,132	-	0.39	-
SMR-CCS	25	1,275,672	-	86,030	-	35,512	-	246.83	2.04	185.88
ATR-CCS	25	1,008,259	-	67,996	-	27,545	-	364.75	4.00	174.71

Salt Cavern Storage	30	-	38,100	-	2,339	-	-	-	0.39	-

Table S12. Power generation CO_2 emissions and CO_2 captured based on a natural gas fuel emission factor of 0.053 tCO_2 /MMBtu [6].

Technology	Fuel combustion emissions (tCO_2 /MWh)	CO_2 capture rate (%)	CO_2 captured (tCO_2 /MWh)
Natural Gas (CC)	0.34	-	-
Natural Gas (CC CCS)	0.04	90.0	0.34
Natural Gas (CT)	0.52	-	-

Table S13. Hydrogen production CO_2 emissions and CO_2 captured based on a natural gas fuel emission factor of 0.053 tCO_2 /MMBtu [6].

Technology	Fuel combustion emissions (tCO_2/tH_2)	CO_2 capture rate (%)	CO_2 captured (tCO_2/tH_2)
SMR-CCS	0.5	96.2	12.6
ATR-CCS	1.1	94.5	18.3

Table S14. Cost and assumptions for DAC technologies obtained from NETL techno-economic study [7, 8]. NGCC = Natural gas combined cycle power plant, CCS = CO_2 capture and storage. DAC technologies with NGCC CCS are self-sufficient by producing electricity in a built-in NGCC unit and capturing natural gas combustion emissions using CCS. In the case of solvent DAC (with NGCC CCS), excess electricity is exported to grid. Investment and fixed operation and maintenance (FOM) costs are based on DAC input CO2 capacity (tonne CO2/h). A discount rate of 4.5% is used to annualize investment costs and costs are converted to 2019 dollars.

Technology	Lifetime (year)	Investment cost ($/($tCO_2$/h))	Annualized CAPEX ($/($tCO_2$)/h)/y)	Fixed operation and maintenance (FOM) cost ($/($tCO_2$/h)/y)	Variable operation and maintenance (VOM) cost ($/$tCO_2$)	Electricity input (MWh/tCO_2)	Natural gas input (MMBtu/tCO_2)
Solvent DAC (with NGCC CCS)	30	14,006,823	859,900	379,948	58	-0.13	10.40
Sorbent DAC (with NGCC CCS)	30	34,789,967	2,135,810	1,180,100	61	-	22.64
Sorbent DAC (Electricity)	30	15,302,494	939,444	747,324	22	4.38	-

Table S15. DAC with built-in NGCC CCS CO_2 emissions and CO_2 captured based on a natural gas fuel emission factor of 0.053 tCO_2 /MMBtu [6]. Deployment of DAC with NGCC CCS will lead to additional CO_2 being stored due to CO_2 capture of NGCC emissions.

Technology	NGCC combustion emissions (tCO_2 /tCO_2 captured by DAC)	NGCC CO_2 capture rate (%)	NGCC CO_2 captured (tCO_2 /tCO_2 captured by DAC)
Solvent DAC (with NGCC CCS)	0.01	99	0.55
Sorbent DAC (with NGCC CCS)	0.12	90	1.08

Table S16. Cost and assumptions for BECCS technologies obtained from Net-Zero America study assumptions [9]. Units of $/MW and MWh/t Biomass for BECCS H_2 are converted based on lower heating value of H_2. A discount rate of 4.5% is used to annualize investment costs and costs are converted to 2019 dollars.

Technology	Lifetime (year)	Investment cost ($/MW)	Annualized Investment cost ($/MW/y)	Fixed operation and maintenance (FOM) cost ($/MW/year)	Variable operation and maintenance (VOM) cost ($/MWh)	CO_2 removal (tCO_2 /MWh)	Bioenergy Yield (MWh/t Biomass)
BECCS Electricity	30	6,843,960	420,161	116,640	25.92	1.07	1,657
BECCS H_2	30	3,317,269	203,836	22,995	14.71	0.62	2,612

Table S17. H2 pipeline data obtained from Hydrogen Delivery Scenario Analysis Model (HDSAM) v2 from Argonne National Laboratory for 100 km pipelines with 20 tonne H2 /h capacity [10]. *HDSAM also provided the pipeline loss fraction, as well as the investment and operational costs, and energy requirements of 2 required compressors per 100 km that were included in the model inputs. This translates into an investment cost of $2214/MW-mile. Discount rate for annualization is 4.5%. Unless otherwise reported, all costs are in 2019 dollars. Candidate pipelines paths (i.e. regional source-sink pairs) and pipeline lengths are the same as power transmission network shown in Table S9.*

Technology	Lifetime	Pipeline Investment Cost ($/MW H_2-mile)	Pipeline Annualized Investment Cost ($/MW H_2-mile/y)
H_2 Pipeline	30	2,214	136

Table S18. CO2 pipeline cost assumptions obtained from 2019 National Academies report on negative emissions technologies [11]. *The report also included the pipeline loss fraction, as well as operational costs and energy requirement of 3 required pumps per 10 miles that were included in the model inputs. We assumed that investment cost of pumps is negligible. The total number of H2 and CO2 pipes (modeled as a continuous variable) along each line will be determined by the model based on the capacity requirement of H2 and CO2 transportation respectively. Discount rate for annualization is 4.5%. Unless otherwise reported, all costs are in 2019 dollars. Candidate pipelines paths (i.e. regional source-sink pairs) and pipeline lengths are the same as the power transmission network shown in Table S9.*

Technology	CO2 throughput (Mt CO2/y)	Lifetime	Pipeline Investment Cost ($/mile)	Pipeline Annualized Investment Cost ($/mile/y)
CO_2 Pipeline	10	30	2,650,000	162,688

Table S19. Existing power generator capacity by resource in each region in 2050. The existing generation capacity in 2050 is estimated from capacity in 2021 after removing natural gas capacity that has exceeded their lifetime (40-50 years depending on natural gas plant type). Data obtained from EIA860 and Public Utility Data Liberation using PowerGenome [12]. Plant lifetimes of natural gas generators are based on NREL ReEDS model input assumption [13]. Based on the Inflation Reduction Act (IRA), second lifetime extensions were assumed for existing nuclear power generators [14]. CC = Combined Cycle. CT = Combustion Turbine.

Technology	CA	NW	SW	TX	NCEN	CEN	SE	MIDAT	NE
Conventional Hydroelectric (GW)	8.77	33.92	4.44	0.47	0.46	5.74	11.12	2.81	4.73
Hydroelectric Pumped Storage (GW)	3.94	0.31	0.78	-	2.00	0.94	6.26	5.24	3.21
Natural Gas CC (GW)	19.96	6.83	21.61	38.18	21.53	29.89	79.62	57.82	6.12
Natural Gas CT (GW)	8.32	1.82	6.53	4.92	16.80	13.46	25.62	15.21	1.41
Nuclear (GW)	-	1.16	4.00	5.02	14.35	7.26	35.86	20.21	5.50
Onshore Wind (GW)	6.06	10.85	5.28	24.93	22.12	27.69	-	3.23	3.65
Small Hydroelectric (GW)	0.34	0.73	0.15	0.02	0.48	0.14	0.25	0.26	0.83
Solar Photovoltaic (GW)	12.31	0.54	7.72	0.60	1.26	1.94	8.50	2.97	1.63

Table S20. Regional cost multipliers for investment cost of new technologies according to region as obtained from the electricity market module in EIA's Annual Energy Outlook (AEO) 2021 [15]. CC = Combined Cycle. CT = Combustion Turbine. CCS = CO_2 capture and storage. The regional cost multipliers are applied to the baseline investment costs of each greenfield technology in Table S10 as model inputs for each region to account for regional variations of costs.

Technology	CA	NW	SW	TX	NCEN	CEN	SE	MIDAT	NE
Natural Gas (CC)	1.30	0.98	0.88	0.91	1.07	0.96	0.93	1.09	1.33
Natural Gas (CC-CCS)	1.07	0.95	0.86	0.95	1.02	0.96	0.96	1.01	1.10
Natural Gas (CT)	1.17	0.98	0.86	0.91	1.07	0.96	0.93	1.03	1.22
Nuclear	1.22	1.09	1.02	0.97	1.08	1.02	1.00	1.03	1.19
Solar	1.07	0.99	0.98	0.96	1.01	0.97	0.97	1	1.07
Land Based Wind	1.89	1.11	0.96	0.94	1.12	0.96	1.10	1.18	1.39
Offshore Wind	1.15	1.00	-	-	-	-	-	-	1.00
Battery	1.04	1.03	1.01	1.01	1.00	1.01	1.02	1.00	1.02

Table S21. Regional cost of fuels for power generation based on EIA's AEO 2020 report, with regions mapped to AEO fuel regions using PowerGenome [12, 16].

Fuel Cost	CA	NW	SW	TX	NCEN	CEN	SE	MIDAT	NE
Natural Gas Price ($/MMBtu)	4.82	4.56	3.89	3.93	4.39	4.41	3.68	3.58	4.4
Uranium Price ($/MMBtu)	0.73	0.73	0.73	0.73	0.73	0.73	0.73	0.73	0.73

Table S22. Capacity reserve margin considered for each region based on the planning reserve margin constraint. The data sourced from electric reliability reports from NERC [17]. The planning reserve margin constraint enforces the need to procure "firm" generation capacity in excess of demand by the specified amount (i.e. reserve margin), where firm capacity contribution of each resource is calculated by either derating its installed capacity (in case of thermal plants) or available generation (in case of non-dispatchable resources like renewables and energy storage and flexible demand).

	CA	NW	SW	TX	NCEN	CEN	SE	MIDAT	NE
Capacity Reserve Margin	0.16	0.16	0.15	0.14	0.16	0.12	0.15	0.17	0.16

Table S23. Unit commitment parameters for resources. Parameters from nuclear power generators obtained from [18, 19], existing gas power generators from [12], new gas power generators from [20]. For blue H2 technologies, start cost and minimum up and down time not modeled, and ramping rates are assumed. Nameplate capacity contributes to the capacity reserve margin (CRM) constraint based on the respective derating factor of each technology, except for electrolyzers where its electricity consumption is the contributing factor. More information on the CRM can be found in the MACRO model documentations [21].

Technology	Start cost ($/MW)	Min up time (h)	Min down time (h)	Max hourly ramping rate (% of nameplate capacity)	Min output (% of nameplate capacity)	Max output (% of nameplate capacity)	Derating factor for capacity reserve margin
Existing Natural Gas (CC) Power Generator	89	6	6	64%	10% - 60%	100%	0.93
Existing Natural Gas (CT) Power Generator	116	1	1	64%	12% - 52%	100%	0.93

Existing Nuclear Power Generator	1,000	36	36	25%	50%	100%	0.93
New Natural Gas (CC) Power Generator	61	4	4	100%	30%	100%	0.93
New Natural Gas (CC-CCS) Power Generator	97	4	4	100%	50%	100%	0.93
New Natural Gas (CT) Power Generator	140	-	-	100%	25%	100%	0.93
New Nuclear Power Generator	1,000	36	36	25%	30%	100%	0.93
SMR-CCS H2 Resource	-	-	-	50%	Depend on scenario	90%	-
ATR-CCS Resource	-	-	-	50%	Depend on scenario	90%	-
Electrolyzer	-	-	-	100%	10%	100%	0.95

Table S24. Annual energy demand for power and H_2. Hourly power demand profiles were obtained from projected 2050 state-level demand from NREL's Electrification Futures Study scenario with high electrification and moderate technology advancement [22]. Annual H_2 demands were obtained from projected 2050 state-level H_2 production (includes H_2 utilized for synthetic fuel production) from Princeton University's Net-Zero America high electrification scenario, with the assumption of constant hourly H_2 demand in creating hourly demand profiles [9]. State-level demands of both power and H_2 demand were aggregated into respective regions based on the states within each region.

Energy Demand	CA	NW	SW	TX	NCEN	CEN	SE	MIDAT	NE	Total
Power (EJ/year)	1.31	1.06	1.37	2.03	2.56	2.43	4.36	3.07	1.15	19.34
H_2 (EJ/year)	0.21	0.27	0.29	0.55	1.97	1.59	0.85	0.81	0.43	6.97

Table S25. Weighted regional average annual capacity factor and maximum available capacity expansion of greenfield variable renewable energy (VRE). VRE profiles were quantified using ZEPHYR (Zero-emissions Electricity system Planning with HourlY operational Resolution), a public repository where solar and wind site capacities and variability profiles were generated using historical weather data from 2007-2013 from NREL NSRDB (National Solar Radiation Database) and WTK (WIND Toolkit) [23, 24, 25]. Together with the input of existing transmission line capacity data from NREL ReEDS (Regional Energy Development Model), the ZEPHYR model generates the available land area for solar and wind power development and determines the interconnection cost of each site (spur-line to substation and trunk-line reinforcement to urban areas) [13]. For each state, three bins (supply curves) containing hourly capacity factor, maximum capacity expansion, and interconnection transmission costs each for wind and solar resources were obtained. In ZEPHYR, state-level data was obtained by utilizing 4x4 km grids and translating hourly wind speed and irradiance into capacity factors using data from NSRDB and WTK respectively. National parks, urban areas, mountains, water bodies, and native land were excluded from the available area for greenfield VRE development, The maximum capacity expansion were obtained by multiplying the available area for VRE development by power generation density of 28 MW/km2 in wind and 1.6 MW/km2 in solar sites respectively [26]. For regions with a single state such as CA and TX, all 3 bins were utilized to represent a diverse choice of VRE availability. On the other hand, in regions with multiple states, the first bin of each state within that region was utilized (For example, NE region with 7 states modeled will have 7 bins of solar and wind resources each). The interconnection transmission cost of each bin was added onto the investment cost of greenfield wind and solar technology from NREL ATB in Table S10 as the model inputs. The weighted average capacity factor presented here are based on the 11 weeks model inputs after time domain reduction was performed on the VRE capacity factor profiles of 2007-2013. The capacity factor in each hour of the 11 representative weeks was multiplied by their respective time weight, and weighted average across the total maximum capacity expansion of the bins utilized in each zone.

Region	CA	NW	SW	TX	NCEN	CEN	SE	MIDAT	NE
Weighted Average Capacity Factor									
Solar	0.32	0.24	0.29	0.29	0.22	0.25	0.26	0.23	0.21
Land Based Wind	0.23	0.38	0.39	0.43	0.45	0.46	0.34	0.4	0.43
Maximum Capacity Expansion (GW)									
Solar	5,953	8,913	9,404	18,172	10,908	9,021	8,503	4,328	2,738
Land Based Wind	340	945	736	10,38	811	864	671	338	206

Table S26. CO_2 storage availability, maximum CO_2 injection rate, and cost of CO_2 injection (in 2019 dollars) in each region. CO_2 geological sequestration sites are obtained from NREL ReEDS (Regional Energy Development Model) [13], and aggregated in each region to obtain the total available CO_2 storage capacity along with their respective maximum CO_2 injection rate, with the CO_2 storage capacity divided by 100 to obtain the annual CO_2 storage availability for model input assuming injection is spread over 100 years. The cost of CO_2 injection is obtained as the weighted average cost of CO_2 injection of the sites in each region according to NREL ReEDS.

	CA	NW	SW	TX	NCEN	CEN	SE	MIDAT	NE
CO_2 Storage Availability (Mt CO_2/y)	4,856	278	132	13,787	427	1,163	2,154	3	0
Maximum CO_2 Injection Rate (tCO_2/h)	39,960	14,293	3,671	29,907	90,089	48,914	31,125	1,155	0
Cost of CO_2 Injection ($/t$CO_2$)	13.55	14.72	15	12.6	9.25	12.92	12.26	11.56	0

Table S27. Biomass availability and cost in each region. Herbaceous and woody biomass are quantified using project 2040 biomass availabilities with 1% yield scenario from the 2016 Billion Ton Study [27]. Biomass costs were obtained by linearizing region-level supply curves built from aggregating county-level biomass using delivered cost from 2016 Billion Ton Study in each region, and adding a transportation cost from respective counties to the centroid of the region.

	CA	NW	SW	TX	NCEN	CEN	SE	MIDAT	NE
Herbaceous Biomass Availability (Mt/y)	1.1	0.3	1.6	62.4	99.3	145.7	49.6	10.8	1.4
Herbaceous Biomass Cost ($/t)	111	107	117	108	125	114	124	123	123
Woody Biomass Availability (Mt/y)	2.7	13.3	2.8	10.1	16.1	24.0	44.2	19.2	5.6
Woody Biomass Cost ($/t)	123	144	135	117	123	122	129	105	102

S7. Methods for estimating electricity and H2 prices

Average electricity price of each zone is obtained using the follow equation:

$$Avg.elec.price\ of\ each\ zone\ \left(\frac{\$}{MWh}\right) = \frac{\sum_t \omega_t \times (\lambda_{t,z} + \gamma_{t,z})}{\sum_t \omega_t} \qquad (Eq.S1)$$

Average electricity supply cost for electrolyzers of each zone is obtained using the follow equation:

$$Avg.elec.supply\ cost\ for\ ely.of\ each\ zone\ \left(\frac{\$}{MWh}\right) = \frac{\sum_t \omega_t \times (\lambda_{t,z} + \gamma_{t,z}) \times D_{t,z}^{\mathrm{Ely}}}{\sum_t \omega_t \times D_{t,z}^{\mathrm{Ely}}} \qquad (Eq.S2)$$

System average electricity price is obtained using the follow equation:

$$National\ avg.elec.price\ \left(\frac{\$}{MWh}\right) = \frac{\sum_z \sum_t \omega_t \times (\lambda_{t,z} + \gamma_{t,z}) \times D_{t,z}}{\sum_z \sum_t \omega_t \times D_{t,z}} \qquad (Eq.S3)$$

System average electricity supply cost for electrolyzers is obtained using the follow equation:

$$National\ avg.elec.supply\ cost\ for\ ely.\left(\frac{\$}{MWh}\right) = \frac{\sum_z \sum_t \omega_t \times (\lambda_{t,z} + \gamma_{t,z}) \times D_{t,z}^{\mathrm{Ely}}}{\sum_z \sum_t \omega_t \times D_{t,z}^{\mathrm{Ely}}} \qquad (Eq.S4)$$

System average H2 price is obtained using the follow equation:

$$National\ avg.H2.price\ \left(\frac{\$}{tonne\ H2}\right) = \frac{\sum_z \sum_t \omega_t \times \lambda_{\mathrm{t,z}}^{\mathrm{H2}} \times D_{t,z}^{\mathrm{H2}}}{\sum_z \sum_t \omega_t \times D_{t,z}^{\mathrm{H2}}} \qquad (Eq.S5)$$

Where:

ω_t *is the time weight of representative period t*

$\lambda_{t,z}$ *is the dual variable of the power supply − demand constraint (i.e. energy price) of zone z at period t*

$\gamma_{t,z}$ *is the dual variable of the capacity reserve margin constraint (i.e. capacity price) of zone z at period t*

$D_{t,z}$ *is the total exogenous and endogeneous power demand of zone z at period t*

$D_{t,z}^{\mathrm{Ely}}$ *is the electrolyzer power demand of zone z at period t*

$D_{t,z}^{\mathrm{H2}}$ *is the H2 demand of zone z at period t*

S8. Model implementation details

The energy system model used here, is a linear program (LP) consisting of approximately 250,000 constraints, 50,000 continuous variables. The model is implemented in JuMP 0.21.8 and each instance is solved using Gurobi 10.0 with barrier and primal simplex method without crossover strategy, using 8 CPUs on MIT supercloud [28]. Typical solve times for each model run are around 12 mins or less. The version of the code used for the study is available on the *"H2-Net-Zero"* branch on the MACRO Github repository [21].

References

[1] L. Vimmerstedt, S. Akar, P. Beiter, W. Cole, D. Feldman, P. Kurup, C. Turchi, G. Oladosu, G. Rhodes, C. Augustine, C. Murphy, A. Schleifer, S. Cohen, J. Hoffmann, P. Schwabe, M. Bolinger, B. Mirletz, M. Bannister, D. Stright, and J. Jenkins, "2021 Annual Technology Baseline (ATB) Cost and Performance Data for Electricity Generation Technologies." DOE Open Energy Data Initiative (OEDI); National Renewable Energy Laboratory (NREL), p. 11 files, 2021. doi: 10.25984/1807473.

[2] International Energy Agency (IEA), "The Future of Hydrogen," (Assumption Annex), IEA, Paris, 2019. [Online]. Available: https://www.iea.org/reports/the-future-of-hydrogen.

[3] U.S. Environmental Protection Agency (EPA), "Power Sector Modeling Platform v.6," EPA, Washington, D.C., 2018.

[4] E. Lewis, S. McNaul, M. Jamieson, M. S. Henriksen, H. S. Matthews, J. White, L. Walsh, J. Grove, T. Shultz, T. J. Skone, E. P., and R. Stevens, "Comparison of Commercial, State-of-the-Art, Fossil-Based Hydrogen Production Technologies," DOE/NETL-2022/3241, 1862910, Apr. 2022. doi: 10.2172/1862910.

[5] D. D. Papadias and R. K. Ahluwalia, "Bulk storage of hydrogen," International Journal of Hydrogen Energy, vol. 46, no. 70, pp. 34527–34541, Oct. 2021, doi: 10.1016/j.ijhydene.2021.08.028.

[6] U.S. Environmental Information Administration (EIA), "Carbon Dioxide Emissions Coefficients," EIA, 2023. [Online]. Available: https://www.eia.gov/environment/emissions/co2_vol_mass.php.

[7] J. Valentine, A. Zoelle, S. Homsy, H. Mantripragada, M. Woods, N. Roy, A. Kilstofte, M. Studivan, M. Steutermann, and T. Fout, “Direct Air Capture Case Studies: Sorbent System,” DOE/NETL-2021/2865, 1879535, Jul. 2022. doi: 10.2172/1879535.

[8] J. Valentine, A. Zoelle, S. Homsy, H. Mantripragada, A. Kilstofte, M. Studivan, M. Steutermann, and T. Fout, “Direct Air Capture Case Studies: Solvent System,” DOE/NETL-2021/2864, 1893369, Aug. 2022. doi: 10.2172/1893369.

[9] E. Larson, C. Greig, J. Jenkins, E. Mayfield, A. Pascale, C. Zhang, J. Drossman, R. Williams, S. Pacala, R. Socolow, EJ Baik, R. Birdsey, R. Duke, R. Jones, B. Haley, E. Leslie, K. Paustian, and A. Swan, “Net-Zero America: Potential Pathways, Infrastructure, and Impacts,” Princeton University, Princeton, NJ, Oct. 2021.

[10] M. Mintz, J. Gillette, A. Elgowainy, M. Paster, M. Ringer, D. Brown, and J. Li, “Hydrogen Delivery Scenario Analysis Model for Hydrogen Distribution Options,” Transportation Research Record, vol. 1983, no. 1, pp. 114–120, Jan. 2006, doi: 10.1177/0361198106198300116.

[11] National Academies of Sciences, Engineering, and Medicine, Negative Emissions Technologies and Reliable Sequestration: A Research Agenda. Washington, D.C.: National Academies Press, 2019, p. 25259. doi: 10.17226/25259.

[12] G. Schivley, E. Welty, N. Patankar, A. Jacobson, Q. Xu, A. Manocha, B. Pecora, R. Bhandarkar, and J. D. Jenkins, PowerGenome/PowerGenome: v0.6.1. (Sep. 08, 2023). Zenodo. doi: 10.5281/ZENODO.8329515.

[13] J. Ho, J. Becker, M. Brown, P. Brown, I. Chernyakhovskiy, S. Cohen, W. Cole, S. Corcoran, K. Eurek, W. Frazier, P. Gagnon, N. Gates, D. Greer, P. Jadun, S.Khanal, S. Machen, M. Macmillan, T. Mai, M. Mowers, C. Murphy, A. Rose, A. Schleifer, B. Sergi, D. Steinberg, Y. Sun, and E. Zhou,“Regional Energy Deployment System (ReEDS) Model Documentation (Version 2020),” NREL/TP-6A20-78195, 1788425, MainId:32104, Jun. 2021. doi: 10.2172/1788425.

[14] The White House, "Inflation Reduction Act Guidebook - Clean Energy," [Online]. Available: https://www.whitehouse.gov/cleanenergy/inflation-reduction-act-guidebook/.

[15] U.S. Environmental Information Administration (EIA), “Annual Energy Outlook 2021,” EIA, Washington, D.C., 2021.

[16] U.S. Environmental Information Administration (EIA), “Annual Energy Outlook 2020,” EIA, Washington, D.C., 2020.

[17] U.S. Environmental Protection Agency (EPA), “Documentation for EPA’s Power Sector Modeling Platform v6 Using the Integrated Planning Model,” EPA, Washington, D.C., 2018.

[18] J. D. Jenkins, Z. Zhou, R. Ponciroli, R. B. Vilim, F. Ganda, F. de Sisternes, A. Botterud, “The benefits of nuclear flexibility in power system operations with renewable energy,” Applied Energy, vol. 222, pp. 872–884, Jul. 2018, doi: 10.1016/j.apenergy.2018.03.002.

[19] N. A. Sepulveda, J. D. Jenkins, F. J. De Sisternes, and R. K. Lester, “The Role of Firm Low-Carbon Electricity Resources in Deep Decarbonization of Power Generation,” Joule, vol. 2, no. 11, pp. 2403–2420, Nov. 2018, doi: 10.1016/j.joule.2018.08.006.

[20] MIT Energy Initiative, "The Future of Energy Storage," MITEI, Cambridge, MA, 2022

[21] G. He, D. S. Mallapragada, R. Macdonald, J. W. Law, Y. Shaker, Y. Zhang, A. Cybulsky, S. Chakraborty and M. Giovanniello, DOLPHYN: Decision Optimization for Low-Carbon Power and Hydrogen Networks. (2024). [Online]. Available: https://github.com/macroenergy/Dolphyn.jl

[22] T. Mai, P. Jadun, J. Logan, C. McMillan, M. Muratori, D. Steinberg, L. Vimmerstedt, R. Jones, B. Haley, B. Nelson, C. Murphy, and Y. Sun, “Electrification Futures Study Load Profiles.” National Renewable Energy Laboratory - Data (NREL-DATA), Golden, CO (United States); National Renewable Energy Laboratory, p. 9 files, 2020. doi: 10.7799/1593122.

[23] M. Sengupta, Y. Xie, A. Lopez, A. Habte, G. Maclaurin, and J. Shelby, “The National Solar Radiation Data Base (NSRDB),” Renewable and Sustainable Energy Reviews, vol. 89, pp. 51–60, Jun. 2018, doi: 10.1016/j.rser.2018.03.003.

[24] C. Draxl, A. Clifton, B.-M. Hodge, and J. McCaa, “The Wind Integration National Dataset (WIND) Toolkit,” Applied Energy, vol. 151, pp. 355–366, Aug. 2015, doi: 10.1016/j.apenergy.2015.03.121.

[25] P. Brown, ZEPHYR. (2022). [Online]. Available: https://github.com/patrickbrown4/zephyr.

[26] P. R. Brown and A. Botterud, “The Value of Inter-Regional Coordination and Transmission in Decarbonizing the US Electricity System,” Joule, vol. 5, no. 1, pp. 115–134, Jan. 2021, doi: 10.1016/j.joule.2020.11.013.

[27] U.S. Department of Energy, “2016 Billion-Ton Report: Advancing Domestic Resources for a Thriving Bioeconomy, Volume 1: Economic Availability of Feedstocks,” M. H. Langholtz, B. J. Stokes, and L. M. Eaton (Leads), ORNL/TM-2016/160, 1435342, Jul. 2016. doi: 10.2172/1435342.

[28] A. Reuther, J. Kepner, C. Byun, S. Samsi, W. Arcand, D. Bestor, B. Bergeron, V. Gadepally, M. Houle, M. Hubbell, M. Jones, A. Klein, L. Milechin, J. Mullen, A. Prout, A. Rosa, C. Yee, and P. Michaleas, “Interactive Supercomputing on 40,000 Cores for Machine Learning and Data Analysis,” 2018, doi: 10.48550/ARXIV.1807.07814.
